# Tailored Design of Mesoporous Nanospheres with High Entropic Alloy Sites for Efficient Redox Electrocatalysis

**DOI:** 10.1002/advs.202402518

**Published:** 2024-07-19

**Authors:** Ravi Nandan, Hiroki Nara, Ho Ngoc Nam, Quan Manh Phung, Quynh Phuong Ngo, Jongbeom Na, Joel Henzie, Yusuke Yamauchi

**Affiliations:** ^1^ Research Center for Materials Nanoarchitectonics National Institute for Materials Science (NIMS) 1‐1 Namiki Tsukuba Ibaraki 305‐0044 Japan; ^2^ Waseda Research Institute for Science and Engineering Waseda University 3‐4‐1 Okubo Shinjuku Tokyo 169‐8555 Japan; ^3^ Department of Materials Process Engineering Graduate School of Engineering Nagoya University Nagoya 464‐8603 Japan; ^4^ Department of Chemistry Graduate School of Science Nagoya University Furo‐cho, Chikusa‐ku Nagoya 464‐8602 Japan; ^5^ Institute of Transformative Bio‐Molecules (WPI‐ITbM) Nagoya University Furo‐cho, Chikusa‐ku Nagoya 464‐8601 Japan; ^6^ Materials Architecturing Research Center Korea Institute of Science and Technology (KIST) 5, Hwarang‐ro 14‐gil, Seongbuk‐gu Seoul 02792 Republic of Korea; ^7^ KHU‐KIST Department of Converging Science and Technology Kyung Hee University Seoul 02447 Republic of Korea; ^8^ School of Chemical Engineering and Australian Institute for Bioengineering and Nanotechnology (AIBN) The University of Queensland Brisbane QLD 4072 Australia; ^9^ Department of Plant & Environmental New Resources Kyung Hee University 1732, Deogyeong‐daero, Giheung‐gu Yongin‐si Gyeonggi‐do 17104 Republic of Korea

**Keywords:** high entropy alloys, hydrogen evolution, hydrogen oxidation, mesoporous nanospheres, Mott–Schottky

## Abstract

High Entropy Alloys (HEAs) are a versatile material with unique properties, tailored for various applications. They enable pH‐sensitive electrocatalytic transformations like hydrogen evolution reaction (HER) and hydrogen oxidation reactions (HOR) in alkaline media. Mesoporous nanostructures with high surface area are preferred for these electrochemical reactions, but designing mesoporous HEA sis challenging. To overcome this challenge, a low‐temperature triblock copolymer‐assisted wet‐chemical approach is developed to produce mesoporous HEA nanospheres composed of PtPdRuMoNi systems with sufficient entropic mixing. Owing to active sites with inherent entropic effect, mesoporous features, and increased accessibility, optimized HEA nanospheres promote strong HER/HOR performance in alkaline medium. At 30 mV nominal overpotential, it exhibits a mass activity of ≈167 (HER) and 151 A g_Pt_^−1^ (HOR), far exceeding commercial Pt‐C electrocatalysts (34 and 48 A g_Pt_^−1^) and many recently reported various alloys. The Mott‐Schottky analysis reveals HEA nanospheres inherit high charge carrier density, positive flat band potential, and smaller charge transfer barrier, resulting in better activity and faster kinetics. This micelle‐assisted synthetic enable the exploration of the compositional and configurational spaces of HEAs at relatively low temperature, while simultaneously facilitating the introduction of mesoporous nanostructures for a wide range of catalytic applications.

## Graphical abstract

A micelle‐assisted wet‐chemical method is used to design and synthesize mesoporous high‐entropy alloy nanospheres at low temperatures. By adjusting reaction conditions, the structure and morphology of these nanospheres can be tailored, resulting in a crystalline state akin to that of a single element. The optimized nanospheres offer new insights into electrochemical catalytic reactions, exhibiting remarkably fast kinetics.

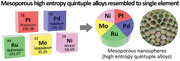

## 1 Introduction

Multi‐metallic nanomaterials have gained significant attention due to their diverse compositional and configurational possibilities.^[^ ^1^, ^2^, ^3^ ^]^ The inherent heterogeneity allows for increased functionality owing to multi‐elemental synergy, making them desirable for various applications including energy storage and conversion, biomedicine, catalysis, sensing, electronics, and photonics.^[^ ^4^, ^5^, ^6^, ^7^, ^8^ ^]^ The recent introduction of high entropy alloy (HEA) nanostructures incorporating five or more principal elements has expanded the multi‐metallic material library, creating unique opportunities to explore and optimize the structure‐property correlation for targeted complex applications.^[^ ^2^, ^3^, ^5^, ^9^, ^10^, ^11^, ^12^, ^13^, ^14^, ^15^ ^]^ The higher configurational entropy in HEA systems overrides the enthalpy penalties, preventing phase segregation by boosting the elemental solubility and resulting in higher phase purity.^[^ ^3^, ^16^ ^]^ Achieving phase purity in HEA results in the confinement of multiple atomic species into the same lattice through entropic contributions.^[^ ^9^, ^17^ ^]^ Phase purity confers two significant benefits: i) the differing sizes of the atomic species cause pronounced lattice distortion, which not only lowers the overall system energy but also slows down interatomic diffusion, resulting in higher overall structural stability.^[^ ^17^ ^]^ Besides, lattice distortion affects the local interatomic interactions, which regulate the adsorption energies of active sites.^[^ ^5^, ^9^, ^17^, ^18^ ^]^ And ii), the confinement of multiple atomic species generates “cocktail effects” that broaden the catalytic range and active site ability beyond what can be realized with monometallic or traditional alloying systems.^[^ ^5^, ^9^, ^17^, ^18^ ^]^

The oxygen evolution reaction (OER)/oxygen reduction reaction (ORR) is a complex multi‐electron transfer process that plays an essential role in electrochemical energy devices like water electrolyzers/fuel cells.^[^ ^19^, ^20^, ^21^, ^22^, ^23^ ^]^ It is essential to note that this process is more favorable in alkaline mediums, while the complementary half‐cell reaction, hydrogen evolution reaction (HER)/hydrogen oxidation reaction (HOR), is more kinetically favorable in acidic mediums.^[^ ^20^, ^24^, ^25^, ^26^, ^27^ ^]^ Unfortunately, integrating these reactions in the same electrochemical device presents practical difficulties, which makes it challenging for electrolyzers and fuel cells to function efficiently. It is also worth noting that when it comes to HER/HOR, Pt/C‐based electrocatalysts are considered state‐of‐the‐art in acidic mediums.^[^ ^26^, ^27^ ^]^ However, they fall short when used in alkaline conditions.^[^ ^26^, ^27^ ^]^ This is because the nature of the intermediate species as well as their concomitant adsorption energy changes depending on the pH of the overall reaction, and the mono‐metallic or traditional alloying systems have active sites that only offer discrete adsorption energy choices.^[^ ^9^, ^24^, ^26^, ^27^ ^]^ They cannot effectively counter the changes in a dynamic reaction environment, leading to overall decreased efficiency of electrocatalysts. This, in turn, requires higher energy consumption in electrolyzers and reduced working/operating voltage (open circuit voltage) windows in fuel cells. HEAs are a potential strategy to address the above limitations.^[^ ^9^, ^17^, ^28^ ^]^ This approach leverages four critical effects: high entropy, cocktail, sluggish interatomic diffusion, and lattice distortion, leading to a near‐continuous range of adsorption energies.^[^ ^9^, ^17^, ^28^ ^]^ HEA sites are expected to offer an expanded catalytic range and greater tolerance toward variations in reaction pH without compromising catalytic efficacy.^[^ ^9^, ^18^ ^]^

In general, electrochemical reactions occur mainly on catalytic surfaces, and nanoparticles (≤10 nm) are favorable due to their high surface‐to‐volume ratio, but they are susceptible to dissolution and agglomeration during aggressive operating conditions.^[^ ^10^, ^29^, ^30^ ^]^ These effects combine to reduce the longevity and overall effectiveness of electrocatalysts. Additional catalytic support platforms are needed to induce stability; however, they may decrease the specific capacity of the catalysts, trigger diffusion limitation, and complicate material synthesis.^[^ ^31^ ^]^ These issues can be addressed by adopting the mesoporous approach in catalyst design. Mesoporous architectures enhance the effective surface area, exposing more active atomic sites to the environment while increasing the effective utilization of materials. At the same time, porous channels boost the effective transportation of reactant/intermediate species.^[^ ^4^, ^5^, ^32^, ^33^, ^34^ ^]^ Adopting the high entropic alloying strategy with embedded mesoporous features has the potential to open a new avenue for the rational design and development of next‐generation electrocatalysts. However, current synthetic approaches like carbothermal, electrosynthesis, solvothermal pyrolysis, mechanical milling, reactive sputter deposition, and laser ablation have limited ability to controllably impose mesoporosity in HEA systems.^[^ ^10^, ^12^, ^17^, ^28^, ^35^, ^36^, ^37^, ^38^, ^39^ ^]^ Although post‐synthesis acid leaching has effectively created meso‐porosity, it leads to material loss and adds complexity to the synthesis and handling process. An alternative approach could involve exploring a wet chemical synthesis protocol in combination with soft templating methods to avoid material loss.^[^ ^40^ ^]^

**Figure 1:**
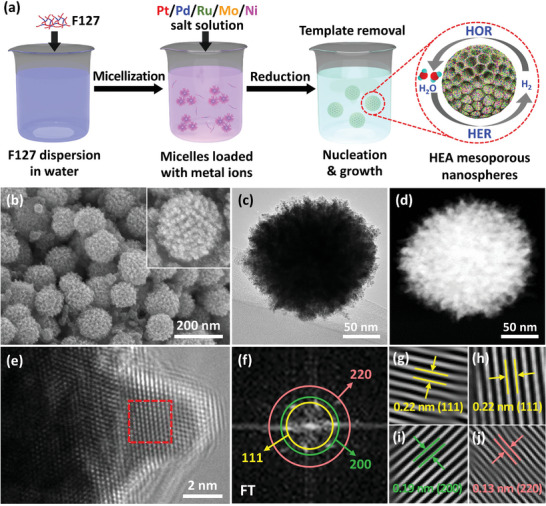
a) Schematic illustration of mesoporous PtPdRuMoNi HEA nanospheres formation using a one‐pot wet chemical reduction process for overall hydrogen evolution and oxidation reactions in alkaline media. The representative b) SEM, c) TEM, and d) HAADF‐STEM images of HEA10 (mesoporous PtPdRuMoNi HEA nanospheres). The associated e) HRTEM image, f) FT pattern, deduced from (e), g–j) corresponding set of planes and concomitant interplanar spacing, deduced from (e) and (f) for HEA10.

In this work, we successfully developed an effective design protocol for creating mesoporous HEA nanospheres of PtPdRuMoNi. This is achieved using a single‐pot wet chemical strategy that utilizes the soft templating feature of Pluronic F‐127 (F127), a triblock copolymer (as shown in **Figure** **1**a) with a polypropylene glycol as the central hydrophobic block flanked with two hydrophilic blocks of polyethylene glycol (PEG). By adjusting conditions including the solvent composition and reaction time, the morphology and reactivity of the final HEA systems are tuned effectively. Importantly, the overall process does not require/demand any sophisticated and dedicated instrumentation facility and hence is immediately scalable. The optimized mesoporous PtPdRuMoNi nanosphere demonstrates its superior catalytic efficiency through electrocatalytic hydrogen evolution and oxidation studies, HER and HOR, in an otherwise kinetically less favored alkaline medium. At a nominal overpotential of 30 mV, the PtPdRuMoNi nanosphere exhibits a specific activity of approximately 167 (HER) and 151 A g_Pt_^−1^ (HOR), which is significantly much higher than that of state‐of‐the‐art Pt‐C‐based electrocatalysts (34 and 48 A g_Pt_^−1^, respectively) and various other recently reported multi‐metallic systems (Table S1, Supporting Information). The computational insights demonstrate the high entropic alloying effect of PtPdRuMoNi nanospheres, resulting in an increased inherent catalytic ability, as evidenced by its higher exchange current density of 2.83 mA cm^−2^ compared to Pt‐C (1.67 mA cm^−2^), an intrinsic material property. The mesoporous structure of the PtPdRuMoNi nanosphere further facilitates the exposure of numerous HEA sites, enabling the migration of reacting species and contributing to its excellent catalytic performance. The methodology used to design the PtPdRuMoNi nanosphere is simple and effective for developing a variety of catalytically active mesoporous HEA nanostructures and can be further explored to develop a wide range of mesoporous HEA systems for diverse fields of applications.

## 2 Results and Discussion

### 2.1 Structural Studies of Mesoporous HEA Nanospheres

The process for designing mesoporous PtPdRuMoNi HEA nanospheres is illustrated in Figure 1a. This result is achieved through a simple one‐pot wet chemical reduction process that utilizes the F‐127 triblock copolymer as a pore‐instituting agent. F‐127 consists of a hydrophobic block at the center, flanked by two hydrophilic blocks. When the copolymer interacts with an aqueous medium such as water, it forms micelles with the hydrophobic branch as the core.

**Figure 2:**
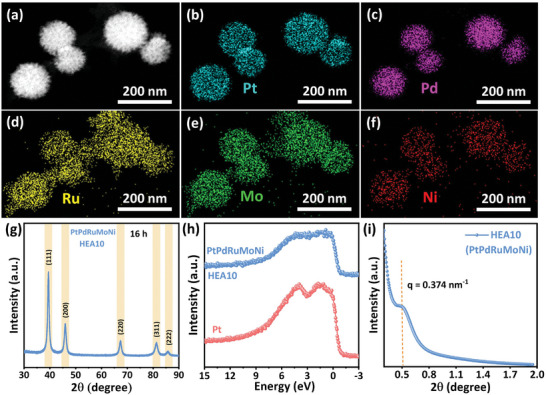
a) HAADF‐STEM image, b–f) corresponding elemental distribution patterns of Pt, Pd, Ru Mo, and Ni, respectively. g) The XRD pattern of HEA10. h) The HAXPES spectrum showing the valance band of HEA10 and Pt using a Cr Kα source. i) The SAXS pattern of HEA10 mesoporous nanospheres. (The scattering vector *q* for SAXS is given by *q* = 4π sin*θ*/*λ*, where *λ* is the wavelength of X‐ray radiation).

The dynamic light scattering (DLS) study reveals that the mean diameter of the micelles is ≈14 nm, as shown in Figure S1 (Supporting Information). The hydrophilic exterior surface of the micelles can accommodate metal ion complexes through either hydrogen bonding or electrostatic interactions, depending on the number of water molecules present in the coordination sphere of the metal ion complexes in an aqueous solution.^[^ ^33^, ^41^, ^42^, ^43^ ^]^ To probe the possible interaction of metal ions with F127‐based micelles, if any, we measured the UV–vis absorption spectra of metal ions containing precursor solution with and without copolymer micelles (Figure S2, Supporting Information). The absorption spectra of the respective metal salts containing solutions exhibit characteristic typical absorption peaks. Interestingly, the metal salt solution with micelles exhibit similar peaks with a noticeable hyperchromic effect. This hyperchromic effect, where the absorption spectra shifted to increased absorbance intensity values without affecting the position of the peaks, indicates an intimate interaction between metal ion complexes and the micelles in the solution (please see the Supporting Information for details).^[^ ^33^ ^]^ F127 polymer was first dissolved entirely in water, creating a transparent solution containing micelles, as evidenced by the Tyndall effect (Figure S3, Supporting Information). Afterward, aqueous metal salt solutions of Ni, Mo, Ru, Pd, Pt, and a reducing agent, L‐ascorbic acid (L‐AA), were added in a controlled manner. The HCl solution was used to regulate the L‐AA‐reducing power.^[^ ^44^ ^]^ The reaction solution was then placed in an oil bath at 95 °C for varying reaction times. The final product, mesoporous PtPdRuMoNi HEA nanospheres, was collected using centrifugation and treated with multiple washes of acetone/ethanol and water to remove the polymeric micelles before undergoing structural characterization. The mesoporous HEA nanospheres are named HEAX, where X represents the individual metal salt concentration used for the synthesis. For example, HEA10 indicates a concentration of 10 mm for each metal salt solution of Pt/Pd/Ru/Mo/Ni used during synthesis. The representative scanning electron microscopic (SEM) image (Figure 1b) of HEA10 shows spherical nanospheres with mesoporous exteriors. This is further confirmed in the bright field transmission electron microscopic (TEM) and the high angle annular dark field (HAADF) scanning TEM (STEM) images (Figure 1c,d) due to contrast differences. The high‐resolution TEM (HRTEM) image near the edges (Figure 1e) suggests a crystalline nature with visible lattice fringes. A randomly selected area in the HRTEM image, indicated by the red box (Figure 1e), was processed to generate a fast Fourier transform (FT) pattern, as shown in Figure 1f. The spots in the FT pattern indicate the presence of (111), (200), and (220) sets of planes corresponding to the face‐centered cubic (fcc) crystal arrangement.^[^ ^45^ ^]^ The inverse FT (IFT) processing was used to obtain the lattice fringes and concomitant interplanar spacings associated with (111), (200), and (220) sets of planes (Figure 1g–j). The interplanar spacing for (111), (200), and (220) sets of planes were found to be 0.22, 0.19, and 0.13 nm, respectively (Figure 1g–j), similar to standard fcc‐phase Pt systems.^[^ ^45^, ^46^ ^]^ The overall TEM study suggests that these PtPdRuMoNi nanospheres are mesoporous and crystalized in the fcc phase. Subsequently, we used energy dispersive spectroscopy (EDS) study with HAADF‐STEM to analyze elemental distribution in the HEA10 system. The study reveals that the randomly selected mesoporous HEA10 nanospheres (**Figure** **2**a) contained metallic Pt, Pd, Ru, Mo, and Ni, which are distributed throughout the structure (Figure 2b–f). This observation is further confirmed in a STEM‐EDS line scan of a single particle (Figure S4, Supporting Information). The inductive coupled plasma‐optical emission spectroscopy (ICP‐OES) further confirms that the HEA10 mesoporous nanospheres contain Pt, Pd, Ru, Mo, and Ni metals (Table S2, Supporting Information). The combined data suggests that although the starting precursor solution contains equivalent atomic ratios, the final HEA mesoporous nanospheres are comparatively rich in Pt and Pd atoms likely due to the favorable reduction kinetics of Pt and Pd metal ions among PtPdRuMoNi elemental grouping. The estimated configurational entropy (Δ*S*_mix_) based on ICP‐OES measurement for HEA10 is 1.5*R*, making them HEA materials.^[^ ^2^ ^]^

The combined evaluation of elemental mapping and line scanning profile suggests that though all five elements are distributed throughout the HEA10 nanospheres, the core seems to be rich in palladium, while the exterior is rich in platinum. This infers that, though the standard reduction potential of Pt complexes ([PtCl_4_]^2−^/Pt ≈ +0.76 V vs SHE) is higher than Pd complexes ([PdCl_4_]^2−^/Pd ≈ +0.59 V vs SHE), the process starts with the preferable reduction of Pd species. This anomalous behavior has been observed previously, even in a bimetallic system of PtPd‐based nanostructures where the core was rich in Pd while Pt was preferentially concentrated at the exterior.^[^ ^40^ ^]^ In a multielement co‐reduction process, the reduction of metal species is very complex and cannot be governed solely by their standard reduction potentials found in the reference literature.^[^ ^44^ ^]^ The reduction kinetics become more complicated due to the presence of multiple metal ion complexes, their coordination environment, organic additives, choice of reducing agent, and reaction ambiance.^[^ ^4^, ^44^, ^47^, ^48^ ^]^

To further understand the high entropy alloying feature of active sites in mesoporous HEA10 nanospheres, we have employed a surface‐sensitive probing technique, X‐ray photoelectron spectroscopy (XPS). The intense peaks associated with Pt, Pd, Ru, Mo, and Ni moieties (Figure S5, Supporting Information) suggest the multi‐elemental character of the HEA10 exterior. In high entropy alloying systems, the inherent heterogeneity and cocktail effects cause very complex interatomic interaction and hybridizations between the neighboring atoms, which modify the valance state giving rise to the core level shift.^[^ ^9^, ^17^ ^]^ Hence, we note the shift of ≈0.5 eV for Pt^0^ 4*f*_7/2_ peak in HEA10 toward the lower energy side compared to standard monometallic Pt systems (Figure S5b, Supporting Information). This observation suggests that the interaction of Pt with other neighboring heteroatoms results in an electron transfer from other metallic moieties to Pt. Pt is more electronegative than Pd, Ru, Mo, and Ni, indicating the electron‐withdrawing tendency of Pt during interatomic neighboring interactions, which could lead to regulating the binding energy (adsorption/desorption) of reactant/intermediate species.^[^ ^5^, ^11^, ^17^, ^18^, ^45^, ^49^, ^50^, ^51^ ^]^ The wave functions of Pt 4*f* and Pt 5*d* levels overlap significantly, suggesting the increase in electron density, which can cause an downward shift of the *d*‐band center, thus modifying the adsorption/desorption energy as the difference between the *d*‐band center and Fermi level used as a matrix of binding energies for reactant species.^[^ ^45^, ^51^ ^]^ Moreover, the Pt‐rich exterior with mesopores and intrinsically supported with high entropy alloying features is expected to benefit our aimed application of HER and HOR in an alkaline medium.^[^ ^26^, ^27^ ^]^ To shed some light on the stable solid solution phase of HEA10, its powder X‐ray diffraction (XRD) pattern is shown in Figure 2g, which unveils the fcc‐phase with characteristic fcc peaks centered at 39.4, 46, 67.4, 81.3, and 85.7 degrees correspond to the (111), (200), (220), (311), and (222) sets of planes, respectively.^[^ ^52^ ^]^ The interplanar spacing obtained from XRD peaks using Bragg's law for (111), (200), and (220) sets of planes (Figure 2g) are found to be 0.22, 0.19, and 0.13 nm, respectively, which matches TEM observation (Figure 1g–j). The lattice constant of the mesoporous PtPdRuMoNi HEA10 nanospheres, deduced from the XRD pattern, is 0.393 nm. This lattice constant is larger than the individual lattice constants of the comprising mono‐metals (i.e., Mo (0.315 nm), Ni (0.349 nm), Ru (0.271 nm), Pd (0.385 nm), and Pt (0.391 nm)), which can be attributed to the phase purity of HEA10. The comparatively larger lattice constant can account for the differing sizes of the atomic species getting accommodated in a single unit cell, causing pronounced lattice distortion to lower the overall system energy for global structural stability.^[^ ^17^ ^]^ Thus, the XRD measurements indicate that the PtPdRuMoNi HEA mesoporous nanospheres crystalize predominantly in the fcc crystal lattice system (Figure 2g) which agrees with TEM measurements (Figure 1f–j). The valence band spectrum was examined using hard X‐ray photoelectron spectroscopy (HAXPES) with a Cr Kα source to probe the impact of entropic effects on the electronic structure of the material. The high energy of the Cr Kα source (5.4 keV) enables non‐destructive analysis deeper inside the material, beyond the surface. Figure 2h shows the valence band (VB) spectrum of monometallic Pt system and HEA10. The monometallic Pt‐system exhibits distinct peaks as expected.^[^ ^11^ ^]^ In contrast, the HEA10 spectrum is broad and featureless, characteristic of high entropy alloys due to the complex orbital hybridization that reflects electronic structure modification.^[^ ^11^, ^53^, ^54^ ^]^ The overall diameter of the HEA10 mesoporous nanospheres is ≈125 ± 10 nm with a pore size of 10 to 12 nm. To evaluate the periodicity of the pores, a small‐angle X‐ray scattering (SAXS) pattern was recorded on HEA10, which shows a peak at *q* = 0.374 nm^−1^ corresponding to a pore‐to‐pore distance of ≈16 nm (Figure 2i). The combined structural and morphological studies suggest that we have successfully employed the wet chemical approach with assisted triblock copolymers to design PtPdRuMoNi mesoporous high entropy alloy nanospheres.

### 2.2 Structural and Morphological Optimization Studies

**Figure 3:**
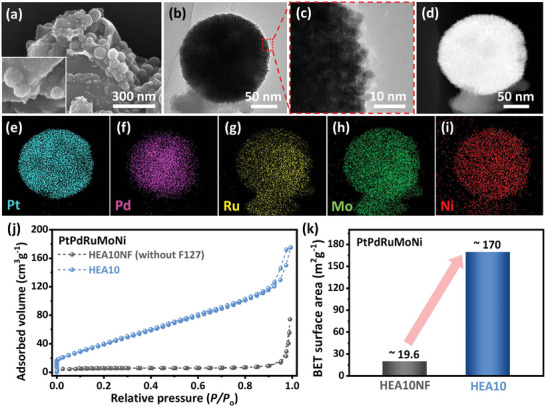
a) SEM and b) bright‐field TEM images of HEA10NF. c) TEM image of a magnified portion of HEA10NF. d) The HAADF‐STEM image and e–i) corresponding elemental distribution patterns in HEA10NF. g) N_2_ adsorption‐desorption isotherms, and j) BET surface areas for HEA10 and HEA10NF.

In the process of creating inorganic nanocrystals through wet chemical synthesis, adding a reducing agent to a solution of metal salt causes nucleation to occur, resulting in a colloidal solution.^[^ ^4^, ^55^, ^56^ ^]^ Subsequently, the nuclei in this solution grow through continuous growth or coalescence, or a combination of both.^[^ ^4^, ^55^, ^56^, ^57^, ^58^, ^59^, ^60^ ^]^ In our study, we examined this process by synthesizing a PtPdRuMoNi HEA system using the same method as for HEA10 mesoporous nanospheres but without the use of F127 copolymer, leading to the creation of HEA10NF (**Figure** **3**, the NF stands for no F127 was used during synthesis). HEA10NF has an agglomerated structure consisting of bulk sheets and nanospheres (Figure 3a). The bright field TEM images (Figure 3b,c) in combination with the HAADF‐STEM image (Figure 3d), show that the structure is made up of individual small nanoparticles (≈4 nm) that coalesced together. This can be explained by the multiple coalescence events that randomly occur to reduce the high surface energy caused by the large surface‐to‐volume ratio and high collision frequency, which drives the ensemble growth of the final structure instead of the individual growth of the small nanoparticles.^[^ ^56^, ^57^, ^58^, ^61^ ^]^

The EDS elemental distribution studies in HAADF‐STEM mode on HEA10NF suggest the distribution of Pt, Pd Ru, Mo, and Ni throughout the structure (Figure 3e–i, Table S2, Supporting Information), like that of in HEA10. This further infers that the use of a copolymer like F127, a soft templating approach, during synthesis brings twofold effects, evident from the morphological appearance of HEA10 and HEA10NF. First, it institutes the porous feature in the final structure (Figures 1b–d and 3a–d), which was manifested from the physicochemical studies of nitrogen adsorption/desorption isotherms and deduced Brunauer–Emmett–Teller (BET) specific surface area (Figure 3j,k, HEA10 ≈ 170 m^2^ g^−1^ and HEA10NF ≈ 19.6 m^2^ g^−1^). Second, the hydrophilic exterior of micelles engages with the metal ions complexes (Figure S2, Supporting Information), thus localizing the nucleation sites. It is also expected to reduce the overall mobility of nuclei and, hence, the collision frequency. This can be reflected in terms of increased nanospheres dispersion as well as the smaller size of HEA10 nanospheres (Figure 1b), while the absence of micelles‐supported synthesis results in the overall structural agglomeration in HEA10NF (Figure 3a).

**Figure 4:**
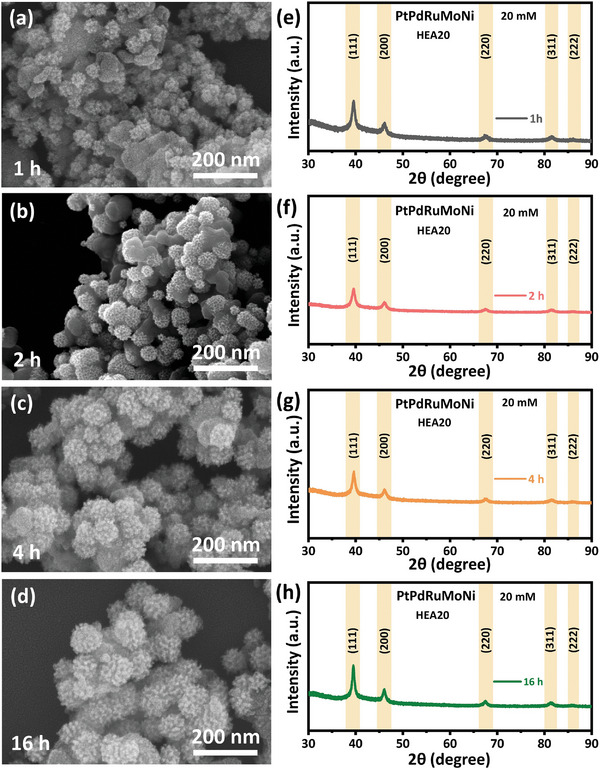
a–d) Time‐dependent SEM studies and concomitant e–h) XRD patterns of HEA20.

We further studied the time dependence and concentration dependence of the synthesis protocol. For over 16 h, we examined how the structure of HEA20 evolved using SEM (**Figure** **4**a–d), XRD (Figure 4e–h), and physisorption measurements (Figure S6, Supporting Information). The associated time‐dependent XRD studies on HEA20 (Figure 4e–h) show the characteristic (111), (200), (220), (311), and (222) fcc peaks matching HEA10 (Figure 2g). No additional diffraction peaks for monometallic/intermetallic or oxides‐based systems are observed in XRD, suggesting that mesoporous HEA20 nanospheres crystallized in predominantly fcc phase. In addition, the physisorption measurements (Figure S6, Supporting Information) show that the effective surface area and pore volume are noticeably high for mesoporous nanospheres obtained for a reaction time of 16 h. The EDS elemental mapping and line scans show that Pt, Pd, Ru, Mo, and Ni are uniformly distributed in the HEA20 mesoporous nanospheres (Figures S7–S9, Supporting Information). Thus, the time‐dependent studies indicate that the optimum reaction time for mesoporous HEA nanospheres is ≈16 h. Furthermore, to study the effect of nucleation on overall structure assembly and growth, the metal salt concentration in precursor solution was varied (1, 2, 5, and 10 mm), keeping other reaction conditions unchanged (i.e., F127 copolymer concentration, reaction time ≈16 h, amount of L‐AA and HCl). Accordingly, the final products were named HEA1, HEA2, HEA5, and HEA10, respectively. In HEA1, the low concentration of metal ions in the precursor solution results in irregular nanostructures with arbitrary sizes (Figure S10a,b, Supporting Information). Based on the XRD pattern, HEA1 is amorphous, however, spherical nano features emerge as the concentration of metal ions in the precursor solution increased from 1 to 2 mm (Figure S10c,d, Supporting Information). A small peak in the XRD pattern at 39.5° indicated overall poor crystallinity. This peak becomes more prominent as the concentration of metal ions increases to 5 mm for HEA5 (Figure S10e,f, Supporting Information). Another peak at 46°, characteristic of the fcc structure, also begins to emerge (Figure S10g, Supporting Information).^[^ ^52^ ^]^ The final structure of HEA5 has more prominent spherical features than HEA1 and HEA2. These experimental studies suggest that low metal ion concentrations in the precursor solution may not produce enough nucleation and collision events, hindering the growth and evolution of the overall structure. The lack of metal ion concentration in the precursor solution and the subsequent nucleation events may adversely affect the crystalline health of the overall structure. Effectively, with a concentration of 10 mm metal ions in the solution, the final structure of HEA10 is well‐dispersed, mesoporous nanospheres with good crystallinity and a predominant single‐phased fcc system (Figures 1 and 2). This methodology can be useful in designing and developing high‐entropy alloy mesoporous systems at mild reaction conditions. These mild reaction conditions are unique compared to typical high‐temperature methods and could thus offer an opportunity to further expand the compositional space of HEA nanostructures.

### 2.3 Electrocatalytic Characterization

The electrochemical hydrogen evolution from water, HER, is sensitive to pH and is sluggish in an alkaline environment.^[^ ^26^, ^27^ ^]^ High pH adversely affects virtually all aspects of HER, including water dissociation, the adsorption/desorption energy of intermediates, and recombination strength to H_2_, making the HER process energy intensive.^[^ ^24^, ^26^, ^27^ ^]^ Similarly, the HOR is an important half‐cell reaction in hydrogen‐based fuel cells and is also kinetically unfavorable in an alkaline media.^[^ ^25^ ^]^ Therefore, designing efficient catalysts for HER/HOR applications in an alkaline medium is crucial.^[^ ^26^ ^]^ High entropy alloys with diverse active surface sites offering near‐continuum adsorption/desorption energies and mesoporous features could be suitable catalysts for HER/HOR in an alkaline medium.^[^ ^1^, ^17^ ^]^ In this study, HER/HOR reactions are used to evaluate the multifunctional electrocatalytic ability and versatility of mesoporous HEA nanospheres in alkaline media. Before electrochemical measurements, the surface physisorption features of HEA10, HEA10NF, and HEA20 were studied using nitrogen adsorption‐desorption isotherms, as these electrochemical processes depend sensitively on the surface area of materials (Figure S11, Supporting Information). The isotherms profiles for HEA10 and HEA20 exhibit an H1 type hysteresis loop and type IV adsorption isotherm, indicating the inherited presence of mesopores with uniform open geometry, which is in good agreement with SEM observations (Figures 1b and 4d).^[^ ^62^ ^]^ The isotherm profile of HEA10NF indicates it is nonporous, matching SEM and TEM observations (Figure 3). The corresponding BET surface area, pore volume, and pore area for HEA10 (≈170 m^2^ g^−1^, 0.231 cc g^−1^, and ≈102 m^2^ g^−1^) are found to be superior to HEA20 (≈148 m^2^ g^−1^, 0.19 cc g^−1^, and ≈74 m^2^ g^−1^) and nonporous HEA10NF (≈20 m^2^ g^−1^, 0.109 cc g^−1^, and ≈8.5 m^2^ g^−1^) (Figure S10, Supporting Information). The values for HEA10NF are markedly lower, illustrating the role of polymeric pore‐directing agents in increasing the effective surface area. Physisorption studies indicate that the HEA10 mesoporous nanospheres have optimal surface features. To further examine these surface features from an electrochemical point of view, we utilized cyclic voltammetry (CV) in non‐Faradic regions, as well as electrochemical impedance spectroscopy (EIS), hydrogen underpotential deposition (HUPD), and carbon monoxide (CO) stripping to evaluate the double‐layer capacitance (*C*_dl_, Figures S12–S15, Supporting Information)).^[^ ^63^ ^]^ This matrix is directly proportional to the effective electrochemical active surface area of the catalyst.^[^ ^63^ ^]^ The experimental findings demonstrate that HEA10 has the highest *C*_dl_ values (Tables S3–S6, Supporting Information) among HEA10, HEA10NF, and HEA20, which aligns with the trend observed in our physisorption studies (Figure S11, Supporting Information). The *C*_dl_ values for Pt‐C and HEA20, as deduced from HUPD studies, are similar (Table S5, Supporting Information), indicating the similar electroactive nature of catalytic sites. This similarity is reflected in the VB spectrum of HEA20, which exhibits peak‐type features similar to those of monometallic Pt system (Figure S16, Supporting Information). This suggests that the high entropic effect in HEA20 is not very prominent in regulating the overall electronic structure. In contrast, the VB spectrum of HEA10 is featureless as demonstrated in Figure 2h. Although HEA10 and HEA20 have similar XRD and STEM EDS data, the low catalytic activity of HEA20 is attributed to minimal entropic effects, as demonstrated in the HAXPES spectrum (Figure S16, Supporting Information).^[^ ^11^ ^]^ These experimental results suggest that HEA10 mesoporous nanospheres are the optimal catalytic material for HER/HOR processes in an alkaline medium. We also included the state‐of‐the‐art commercially available Pt‐C electrocatalyst for HER/HOR processes for comparison.

**Figure 5:**
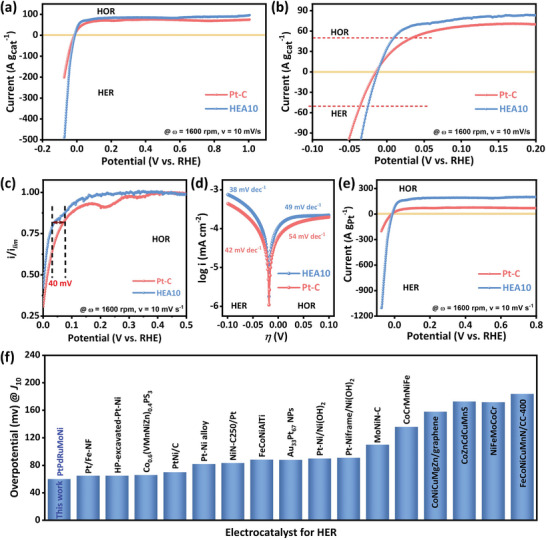
a) LSV‐polarization curves for HER‐HOR on Pt‐C and HEA10 in an alkaline medium (0.1 m KOH). b) The magnified portion of LSV curves from (a) is in the range of ± 100 A g_cat_^−1^. c) HOR polarization curves of Pt‐C and HEA10 normalized to their respective maximum limiting current d) Tafel curves of respective kinetic current densities, e) HER‐HOR polarization curves normalized concerning the Pt weight for Pt‐C and HEA10. f) Overpotential comparative study (@*J*_10_) for HER in an alkaline medium on HEA10 with some of the recently reported HEA, multi‐metallic, and Pt‐based electrocatalysts (tabulated in supporting information Table S1, Supporting Information).

**Figure** **5**a displays the normalized polarization curves concerning the overall catalyst loading for HER‐HOR in an alkaline medium (0.1 M KOH). The results reveal that HEA10 mesoporous nanospheres outperform the Pt‐C electrocatalyst under similar experimental conditions. The plateau region observed in the HOR branch (the positive potential side) is due to the limited transport of H_2_ from the electrolyte solution to the catalyst surface.^[^ ^25^, ^27^ ^]^ To showcase the superiority of HEA10 over Pt‐C, Figure 5b displays a selected portion of the polarization curves (±100 A g_cat_^−1^). HEA10 requires an overpotential of only 9 and 25 mV for HOR and HER, respectively, to achieve a specific current density of 50 A g_cat_^−1^ (Figure 5b). These values are much better than those of Pt‐C (33 mV and 36 mV), indicating the inherently bifunctional active nature of HEA10. To understand the comparative HOR kinetics, the respective HOR polarization curves were normalized with their maximum plateau value current (i.e., diffusion‐limited current density, *I*_lim_, Figure 5c) to neutralize the respective structural/geometrical features of Pt‐C and HEA10. Figure 5c shows the starting point of the HOR plateau, where the overall polarization curves are shifted positively by ≈40 mV for Pt‐C. This indicates that Pt‐C has less favorable reaction kinetics under the same experimental conditions versus HEA10.^[^ ^27^ ^]^ This is further supported by the fact that HEA10 has a higher kinetic current and smaller Tafel slopes than Pt‐C, as shown in Figure 5d. The exchange current value is an intrinsic material property directly correlating to the catalyst's inherent electrochemical active nature, and it is higher for HEA10 (2.83 mA cm^−2^) than for Pt‐C (1.67 mA cm^−2^).^[^ ^26^ ^]^ The improved reaction rate, better kinetic current, low Tafel slopes, and high exchange current for HEA10 can be attributed to its inherent high entropic alloying feature. This is well reflected in its ultrahigh mass activity, which is ≈167 (HER) and 151 A g_Pt_^−1^ (HOR) at a nominal overpotential of 30 mV. These values are significantly higher than those of state‐of‐the‐art Pt‐C‐based electrocatalysts, which are only 34 and 48 A g_Pt_^−1^, respectively (Figure 5e).

The HEA10 material has both high entropy alloying and mesoporous features, making it highly effective for HER‐HOR activities. Nonporous HEA10NF nanospheres show inferior HER performance compared to mesoporous HEA10 nanospheres (Figure S17a, Supporting Information). This is because HEA10NF nanospheres have limited active sites available, as they were prepared without using the micelle templates (Figure 3; Figure S11, Supporting Information). This was confirmed by physisorption and electrochemical studies using CV, EIS, HUPD, and CO‐stripping (Tables S3–S6, Supporting Information). The HEA10 mesoporous nanospheres show better HER activity than many recently reported HEAs, multi‐metallic, and Pt‐based alloys in an alkaline medium (Figure 5f; Table S1, Supporting Information). The potentiometric study also shows that HEA10 can maintain stable performance when subjected to HER for more than 10 h (Figure S17b, Supporting Information). The XRD and SEM studies suggest that HEA10 nanospheres retain their structural and mesoporous features in post‐electrochemical studies (Figure S18, Supporting Information). The double‐layer capacitance, i.e., *C*_dl_, is also nearly comparable (Table S7, Supporting Information), indicating the operational stability of HEA systems. Inspired by promising HER and HOR performance, we also performed the oxygen reduction reaction (ORR) on HEA10 mesoporous nanospheres in an alkaline medium (Figure S19, Supporting Information) under identical reaction conditions. The comparative ORR polarization curves in terms of mass activity (A) on Pt‐C and HEA10 are shown in Figure S19 (Supporting Information). The diffusion‐limited current density at 0.4 V versus RHE for HEA10 (−780 A g_pt_^−1^) is nearly three times higher than Pt‐C (−260 A g_pt_^−1^). Throughout the working potential window of HEA10, peroxide (HO_2_^−^) generation and average electron transfer number (*n*) involved in per oxygen molecule reduction are below 5% and 3.91, respectively. These observations suggest that mesoporous HEA10 nanospheres can function as electrocatalysts in both fuel cells and metal‐air battery systems.

### 2.4 Mott–Schottky (MS) and Computational Analysis

**Figure 6:**
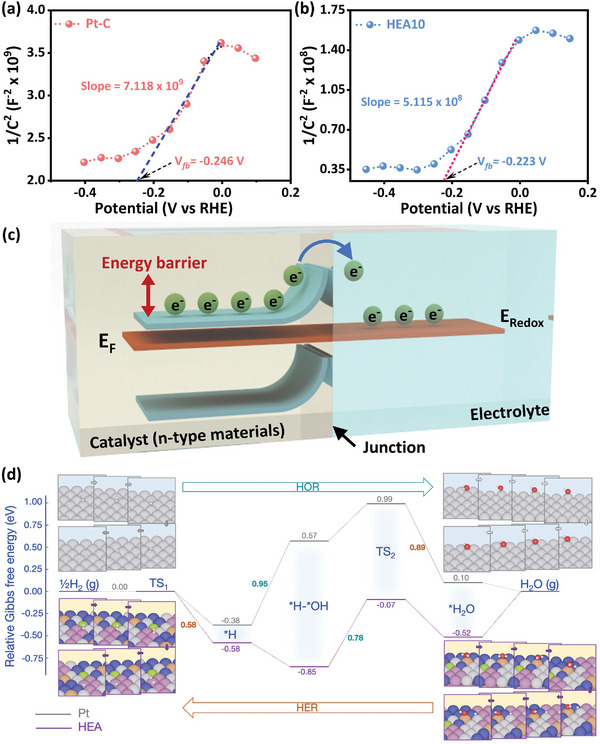
Mott–Schottky plots for a) Pt‐C and b) HEA10 in nitrogen purged 0.1 m KOH aqueous solution (X‐ intercept in MS plot shows the respective flat band potential (*V*_fb_)). c) An illustration showing an upward bending of the band edges for an n‐type material at the electrode‐electrolyte interface at thermodynamical equilibrium. (Flat band potential determines the magnitude of band bending.) d) Calculated Gibbs free energy profiles (in eV) for HER and HOR on the (111) facet of HEA and Pt systems at 0.0 V versus RHE. The brown and teal numbers correspond to the activation free energy of the rate‐determining step of HER and HOR, respectively.

To gain a deeper understanding of electrocatalysts' behavior at the electrode‐electrolyte interface, Mott‐Schottky (MS) analysis was employed in an alkaline medium.^[^ ^64^, ^65^ ^]^ **Figure** **6**a,b show the MS plots (1/*C*_cs_^2^ vs *V*, where *C*_cs_ is the space charge capacitance and *V* is the applied potential) for Pt‐C and HEA10, providing valuable insights into the inherent catalytic ability and electron transfer characteristics of electrocatalysts. The positive slopes of the MS plots indicate that both electrocatalysts are n‐type.^[^ ^64^, ^65^ ^]^ Moreover, the slope of the MS plot is inversely proportional to the charge carrier density, which, in this case, is the electron.^[^ ^65^, ^66^ ^]^ HEA10 displays a slope value that is an order of magnitude smaller than commercially available Pt‐C electrocatalyst (5.12 × 10^8^ compared to 7.12 × 10^9^), indicating a higher charge carrier density in HEA10. In nanostructured electrocatalyst systems, the charge carriers extend into the electrode up to a distance of 100–10 000 Å from the surface, creating a space charge distribution.^[^ ^65^ ^]^ When exposed to electrolytes, these electrocatalysts experience space charge redistribution due to an electron transfer from the electrode to the electrolyte in an n‐type system.^[^ ^64^, ^65^, ^66^ ^]^ This transfer of charge carriers creates a capacitive region known as the space charge double layer, with an associated electric field that facilitates facile charge transfer during reactions.^[^ ^65^ ^]^ This space charge double layer is an intrinsic material property and differs from the interfacial electrode‐electrolyte double layer, which is proportional to the effective surface area or electroactive site density— an extrinsic feature that depends on the material design (Tables S3–S6, Supporting Information).^[^ ^31^, ^65^ ^]^ The Mott–Schottky equation measures the space charge capacitance (*C*_cs_) as a function of applied potential.^[^ ^66^ ^]^ The HEA10, with its high charge carrier density, was expected to exhibit better space charge redistribution, generating a stronger built‐in electric field in the space charge region. The resulting enhanced electronic interactions at the electrode‐electrolyte interface offer optimal adsorption/desorption of intermediate species and comparatively fast charge transfer kinetics.^[^ ^65^, ^66^, ^67^ ^]^ This effect is further supported by the more favorable position of the flat band potential (*V*_fb_, Figure 6a,b) for HEA10.

The magnitude of *V*_fb_ represents the upward band bending for an n‐type system when exposed to electrolyte at thermodynamic equilibrium, hence representing the magnitude of the kinetic barrier for charge transfer, as illustrated in Figure 6c.^[^ ^65^, ^66^, ^67^ ^]^ The *V*_fb_ for HEA10 (−0.223 V, Figure 6c) is found to be positively placed concerning that for commercial Pt‐C (*V*_fb_ = −0.246 V), representing a comparatively reduced energy barrier for better charge transfer owing to smaller upward band bending for HEA10. Overall, the MS analysis of HEA10 shows it has n‐type behavior, high charge carrier density, comparatively minor band bending owing to a more positive flat band potential, and a smaller charge transfer barrier during reactions in an alkaline media versus Pt‐C. These observations show that HEA10 has enhanced catalytic activity and concomitant fast charge transfer kinetics, as evidenced by its superior electrochemical performance (Figure 5; Figures S17 and S18, Supporting Information).

To further support and gain insights into the enhanced electrochemical performance of HEA10, density functional theory (DFT) calculations were performed. In Figure 6d, the HER and HOR processes^[^ ^68^ ^]^ were calculated for the Pt(111) and representative HEA10 surfaces at an electrode potential of 0.0 V versus RHE. We first consider the HER under alkaline conditions, starting with the Volmer step H_2_O + e^−^ + * → *H + OH^−^.^[^ ^69^ ^]^ This consists of three elementary reactions; one can readily observe distinct differences between HEA and Pt surfaces. In the first reaction, H_2_O is adsorbed, which is exergonic on the HEA surface (−0.52 eV) and endergonic on Pt(111) (0.1 eV). The adsorbed H_2_O molecule dissociates into *H and *OH in the second reaction. The activation energy of this reaction is ≈0.9 eV on the Pt(111), while on the HEA surface, it is notably lower (only 0.45 eV). The reduced activation energy on the HEA surface, consistent with the Evans–Polanyi principle, is attributed to its enhanced ability to strongly bind *H and *OH species. This reduction of activation energy, indicative of improved water dissociation, should directly contribute to the higher HER activity observed on the HEA surface. At the end of the Volmer step, the *OH species is converted into OH^−^. This conversion is downhill on Pt(111) by 0.95 eV, whereas on the HEA surface, it goes uphill by 0.27 eV. In the subsequent Tafel step (*H → ½H_2_), the activation free energy on the HEA surface is calculated to be 0.58 eV, whereas the Pt(111) surface exhibits better H_2_ desorption with a slightly smaller activation free energy (0.38 eV). However, for a comprehensive comparison of the catalytic activity between Pt and HEA, it is essential to identify the rate‐determining step (RDS) of the entire Volmer‐Tafel mechanism. On the Pt(111) surface, the RDS is determined to be the water dissociation step, characterized by an activation energy of 0.9 eV, whereas the RDS on the HEA surface is predicted to be the Tafel step with an activation energy of 0.58 eV. Overall, among three factors governing the alkaline HER activity^[^ ^1^ ^]^—*H binding, *OH binding, and water dissociation—the latter appears to explain the superior HER activity observed on the HEA10 surface.

Similarly, we briefly discuss the Gibbs free energy diagram for the HOR (as the reverse process of the HER shown in Figure 6d). Although the HEA surface exhibits a rather high affinity toward hydrogen, its surface also demonstrates good ability in adsorbing OH^−^, which can readily combine with *H to form *H_2_O. Accordingly, this combination step turns out to be the RDS with an activation energy of 0.78 eV. In contrast, the RDS on the Pt(111) surface involves the *H + OH^−^ → *H‐*OH reaction, with an activation free energy of up to 0.95 eV. The lower activation energy on the HEA surface obtained in our calculations is consistent with the observed higher HOR kinetics than Pt‐C‐based electrocatalysts.

## 3 Conclusion

In this study, a wet‐chemical method utilizing a triblock copolymer has been developed to create mesoporous HEA nanospheres consisting of PtPdRuMoNi. The surface electronic structure and overall morphology of these HEA nanospheres can be modulated by varying reaction conditions. The growth of these HEA nanospheres is governed by multiple coalescence events, resulting in ensemble‐driven overall growth of the structure. The study also finds that the crystalline health of the final product depends on the metal ion concentration in the precursor solution. The use of polymeric micelles successfully creates mesopores in the HEA nanospheres, increasing their effective surface area, pore volume, and accessibility to reactant species. The optimized mesoporous HEA nanosphere (HEA10) demonstrates excellent hydrogen evolution and oxidation reaction activities with faster reaction kinetics in an alkaline medium. HEA10 exhibits a mass activity of approximately 167 (HER) and 151 A g_Pt_^−1^ (HOR) at a nominal overpotential of 30 mV, which is significantly higher than that of state‐of‐the‐art Pt‐C‐based electrocatalysts (34 and 48 A g_Pt_^−1^). This high activity is attributed to HEA10's intrinsic catalytic active nature and tendency for faster charge transfer owing to its high entropy alloying features, as revealed by experimental outcomes, MS analysis, and computational insights. Our strategy can open an avenue for copolymers‐assisted design of a wide range of multifunctional mesoporous high entropy nanoalloys with rational morphologies for a diverse range of targeted applications.

## 4 Experimental Section

### Materials

All the chemicals used in the synthesis were received without further purification. The metal salts used were Potassium tetrachloroplatinate(II) (K_2_PtCl_4_, 98%), Sodium tetrachloropalladate(II) (Na_2_PdCl_4_, 98%), Ruthenium(III) chloride hydrate (RuCl_3_.xH_2_O, 99.98%), Molybdenum (V) chloride (MoCl_5_, 95%) and Nickle(II) chloride hexahydrate (NiCl_2_.6H_2_O, 99.9%), purchased from Sigma‐Aldrich. The triblock copolymer Pluronic F‐127 (C_3_H_6_O.C_2_H_4_)_x,_ BioReagent grade), L‐ascorbic acid (C_6_H_8_O_6_, MW. 176.12, Reagent grade), Nafion perfluorinated resin solution (5 wt. %) were purchased from Sigma Aldrich. The carbon black Vulcan (XC‐72R) used during catalyst ink preparation was purchased from The Fuel Cell Store. Ethanol and acetone were purchased from Nacalai Tesque, Inc. For all synthesis, washing, and electrochemical measurements Milli‐Q water with an ionic purity 18.2 MΩ was used.

### Materials Characterization

The structural and morphological observation and analysis were carried out using FE‐SEM+EDX [SU8000] scanning electron microscope (SEM, Hitachi High Technologies/Bruker, model no.‐ SU8000/Quantax FQ5060) and 200 kV field emission transmission electron microscope (TEM, JEM‐2100F1) JEOL Ltd.). Surface elemental composition was probed using X‐ray photoelectron spectroscopic (XPS) analysis device (Quantera SXM) from ULVAC‐PHI Co., Ltd. Monochromatic Al Kα (1486.6 eV) focused X‐ray source was used for excitation. The C 1s binding energy (284.6 eV) was used for calibration. The same instrument was used to obtain valance band (VB) spectra with Cr Kα (5417 eV) focused X‐ray source for excitation. The elemental composition was obtained using an induced binding plasma emission optical analysis device (ICP‐OES, high resolution type) (SPS3520UV‐DD) from Hitachi High‐Tech Science Co., Ltd. The powder X‐ray diffraction patterns were recorded using MiniFlex600 system (X‐ray wavelength: Cu Kα) from Rigaku Co., Ltd. The small‐angle X‐ray scattering (SAXS) pattern was recorded using Rigaku NANO‐Viewer for pore‐to‐pore distance measurement. Nitrogen adsorption–desorption isotherms were obtained using BELSORP‐mini (BEL, Japan) at 77 K, and the specific surface area and porous feature analysis were carried out using Brunauer–Emmett–Teller (BET) and Barrett‒Joyner‒Halenda (BJH) models. Particle size analyzer from Otsuka Electronics Co., Ltd. (Model No. ELSZ‐2000ZS, particle size analysis specification: 0.6 nm to 8 µm) was used to evaluate the average size of micelles dispersed in aqueous medium. The UV–vis spectroscopic system from TECAN (model no.‐ M200 PRO) was used to record the absorption spectra of metal salt solution with and without polymer micelles in an aqueous medium.

### PtPdRuMoNi‐HEA Nanospheres Synthesis

The PtPdRuMoNi high entropy alloys (HEA) nanospheres were synthesized by the assembly of a triblock copolymer (F127) micelles using a wet‐chemical approach. In a typical synthesis, 200 mg of Pluronic F127 triblock copolymer was mixed with aqueous solutions of K_2_PtCl_4_ (10 mm, 6 ml), Na_2_PdCl_4_ (10 mm, 6 ml), RuCl_3_.xH_2_O (10 mm, 6 ml), MoCl_5_ (10 mm, 6 ml), NiCl_2_.6H_2_O (10 mm, 6 ml) under sonication to completely dissolved the F127. Furthermore, 0.8 ml of 6 m HCl was added to this solution. This precursor solution was gently stirred for 30 min for homogeneous mixing and transferred to oil bath kept at 95 °C. After 10 min, 8 mL *L*‐asocrbic acid (*L‐*AA, 0.1 m) solution was carefully introduced into the precursor solution under gentle stirring conditions for 16 h reaction time under reflux. The nonporous PtPdRuMoNi (HEA10NF) was prepared using a similar method without using F127 copolymer. To explore the insights of HEA nanospheres synthesis process, the metal salt solutions concentration (1, 2, 5, 10, 20 mm) and reaction time (1, 2, 4, 16 h) were varied accordingly (Figure 4, main text and Figure S10, Supporting Information). The final product, post‐reaction, was collected by centrifuging (14 000 rpm, 25 min) and washed multiple times with acetone/ethanol/water to remove soft polymer templates.

### Electrochemical Characterization

The high entropy alloys (HEAs) nanospheres were grinned by a mortar. The HEAs (10 mg) were sonicated in 5 ml of hexane before adding 40 mg carbon black (XC‐72) and 30 mL hexane, followed by sonication for 1 h at room temperature. The resulting powder was collected by centrifugation (12 000 rpm) and washed three times with ethanol. The powder was dried in a vacuum oven (DP‐200, Yamato Scientific co., ltd.) at 60 ˚C for 12 h. To prepare the working electrode, 5.0 mg of the carbon‐supported catalysts was dispersed in a 950 µL mixture of DI and isopropanol (1:2 vol, v/v) and a 50 µL of 5 wt.% Nafion solution. The suspension was ultrasonicated for at least 60 min to obtain a homogeneous ink (catalyst concentration of 5 mg mL^−1^). The ink was drop‐casted on a 3 and 4 mm diameter glassy carbon disk electrode for RDE and RRDE, respectively, to obtain the catalyst density of 0.25 mg cm^−2^. An aqueous alkaline solution (0.1 m KOH) media was used to study the hydrogen evolution reaction (HER), hydrogen oxidation reaction (HOR), and oxygen reduction reaction (ORR).

### Computational Details

Plane‐wave DFT calculations employing the Perdew–Burke–Ernzerhof (PBE) function^[^ ^70^ ^]^ were performed with the Vienna Ab initio Simulation Package (VASP) package.^[^ ^71^, ^72^ ^]^ The projector augmented wave (PAW) method^[^ ^73^, ^74^ ^]^ with wave functions expanded to an energy cutoff of 450 eV. Grimme's D3 correction Becke‐Johnson damping function was applied to account for weak dispersion interactions.^[^ ^75^ ^]^ A Gaussian smearing width of 0.2 eV was applied to all calculations. The irreducible Brillouin zone was sampled using a Γ‐centered k‐point mesh of 2 × 2 × 1. The metal surfaces were simulated using a 5 × 5 slab cell consisting of 100 atoms (four atomic layers). The two bottom layers were fixed, whereas the remaining atoms were relaxed with thresholds of 10^−4^ eV and 5 × 10^−2^ eV Å^−1^ for the energy and residual force, respectively. In all calculations, a vacuum of 15 Å along the z‐direction was used to minimize Coulombic interactions with periodic self‐images.

To obtain the Gibbs free energy profile for HER and HOR, the widely used Nørskov's computational hydrogen electrode model was adopted,^[^ ^76^, ^77^, ^78^ ^]^ in which the Gibbs free energy of a proton–electron pair was estimated as *G*(H^+^ + e^−^) = 1/2 *G*(H_2_) and the Gibbs free energy of OH^−^ was calculated as *G*_OH–_ = *G*_H2O_ − 1/2*G*(H_2_). The Gibbs free energy of other reaction intermediates was computed as *G* = *E* + *E*_ZPE_ + *TS* where *E*, *E*_ZPE_, and *S* represent the DFT total energy, zero‐point energy, and vibrational entropy of adsorbates (calculated under standard conditions of *p* = 1 atm and *T* = 298.15 K), respectively.^[^ ^79^ ^]^ Transition states (TS) of H_2_O and H_2_ desorption processes were calculated using the climbing image nudged elastic band (CINEB) method.^[^ ^80^ ^]^ In particular, the activation barrier was defined by the difference between the TS and the corresponding stable configurations of reactants.

## Conflict of Interest

The authors declare no conflict of interest.

## Supporting information

Supporting Information

## Data Availability

Research data are not shared.
