# Supplementary material for: Tailored Design of Mesoporous Nanospheres with High Entropic Alloy Sites for Efficient Redox Electrocatalysis
Source: Adv Sci (Weinh). 2024 Jul 19;11(35):2402518. doi: 10.1002/advs.202402518 (PMC11425213; doi:10.1002/advs.202402518)
Supplement: Supplementary file 1 — Supporting Information [file ADVS-11-2402518-s001.docx]

**Tailored Design of Mesoporous Nanospheres with High Entropic Alloy Sites for Efficient Redox Electrocatalysis**

Ravi Nandan,^a^ Hiroki Nara,^b^ Ho Ngoc Nam,^c^ Quan Manh Phung,^d,e^ Quynh Phuong Ngo,^f^ Jongbeom Na,^f,g,h^* Joel Henzie,^a,^* and Yusuke Yamauchi ^a,c,h,i^*

*^a^ Research Center for Materials Nanoarchitectonics, National Institute for Materials Science (NIMS), 1-1 Namiki, Tsukuba, Ibaraki 305-0044.*

*^b^ Waseda Research Institute for Science and Engineering, Waseda University, 3-4-1 Okubo, Shinjuku Tokyo 169-8555, Japan.*

*^c^ Department of Materials Process Engineering, Graduate School of Engineering, Nagoya University, Nagoya 464–8603, Japan*

*^d^ Department of Chemistry, Graduate School of Science, Nagoya University, Furo-cho, Chikusa-ku, Nagoya 464-8602, Japan.*

*^e^ Institute of Transformative Bio-Molecules (WPI-ITbM), Nagoya University, Furo-cho, Chikusa-ku, Nagoya 464-8601, Japan*

*^f^ Materials Architecturing Research Center, Korea Institute of Science and Technology (KIST), 5, Hwarang-ro 14-gil, Seongbuk-gu, Seoul 02792, Republic of Korea*

*^g^ KHU-KIST Department of Converging Science and Technology, Kyung Hee University, Seoul 02447, Republic of Korea*

*^h^ School of Chemical Engineering and Australian Institute for Bioengineering and Nanotechnology (AIBN), The University of Queensland, Brisbane, QLD 4072, Australia*

*^i^ Department of Plant & Environmental New Resources, Kyung Hee University, 1732, Deogyeong-daero, Giheung-gu, Yongin-si, Gyeonggi-do 17104, South Korea*

Corresponding Author

Email: jongbeom@kist.re.kr (Jongbeom Na, orcid.org/0000-0002-3890-7877)

Email: HENZIE.Joeladam@nims.go.jp (Joel Henzie, orcid.org/0000-0002-9190-2645)

Email: y.yamauchi@uq.edu.au (Yusuke Yamauchi, orcid.org/0000-0001-7854-927X)

**
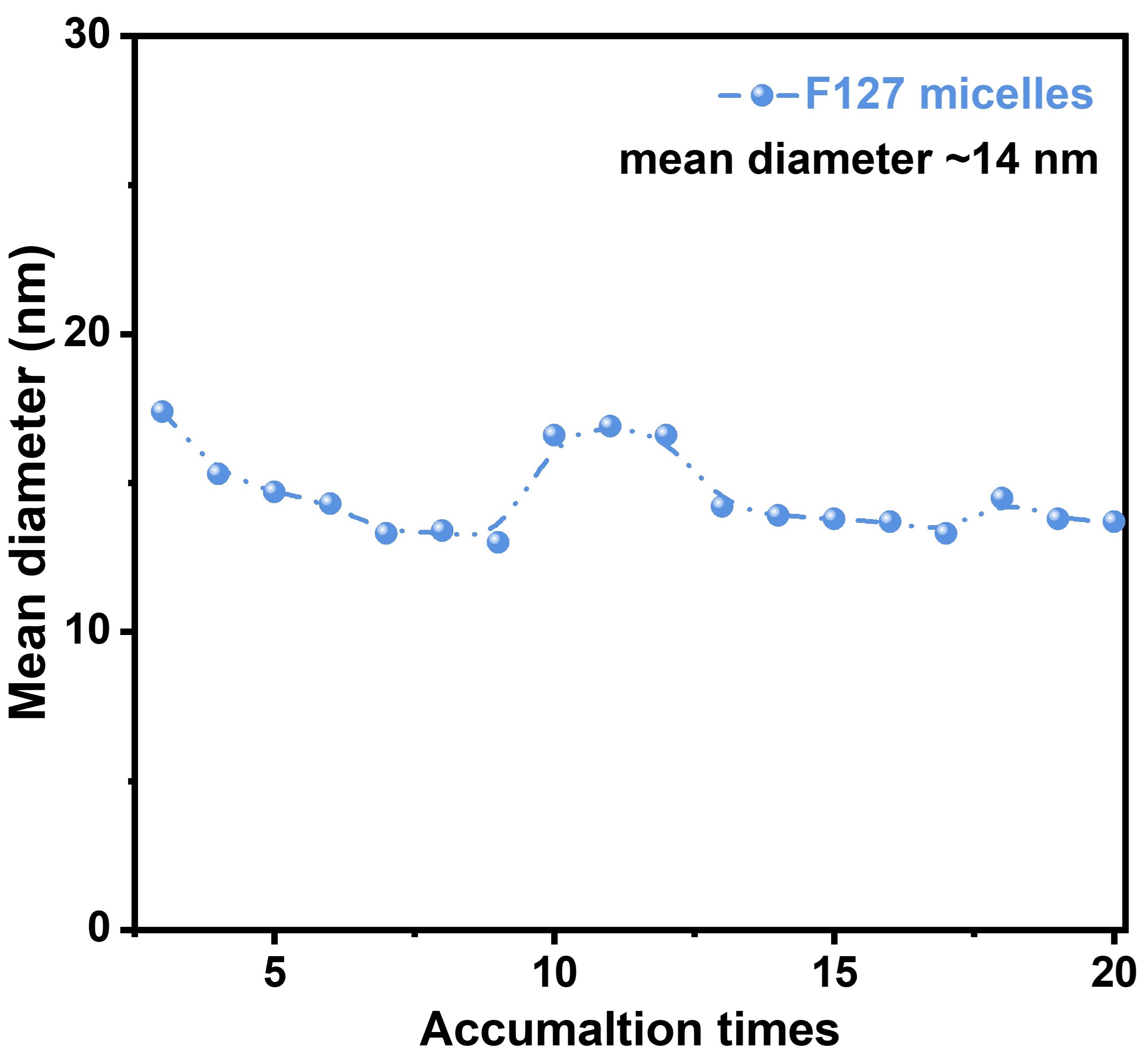
**

**Figure S1.** Mean diameter determination of F127 micelles in DI water using dynamic light scattering process.

**
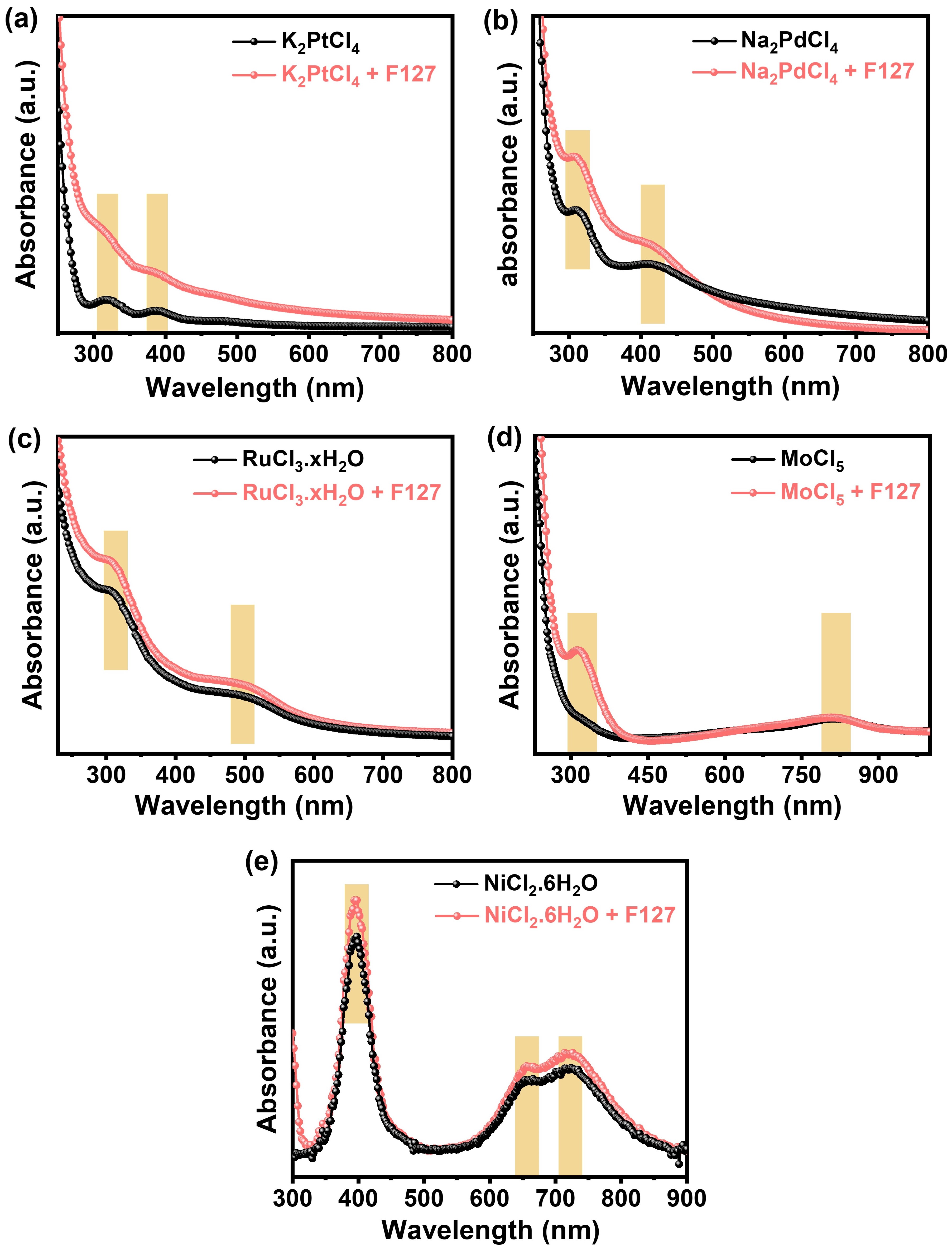
**

**Figure S2.** UV-visible spectra of various salt solutions with and without F127 micelles in aqueous medium.

**
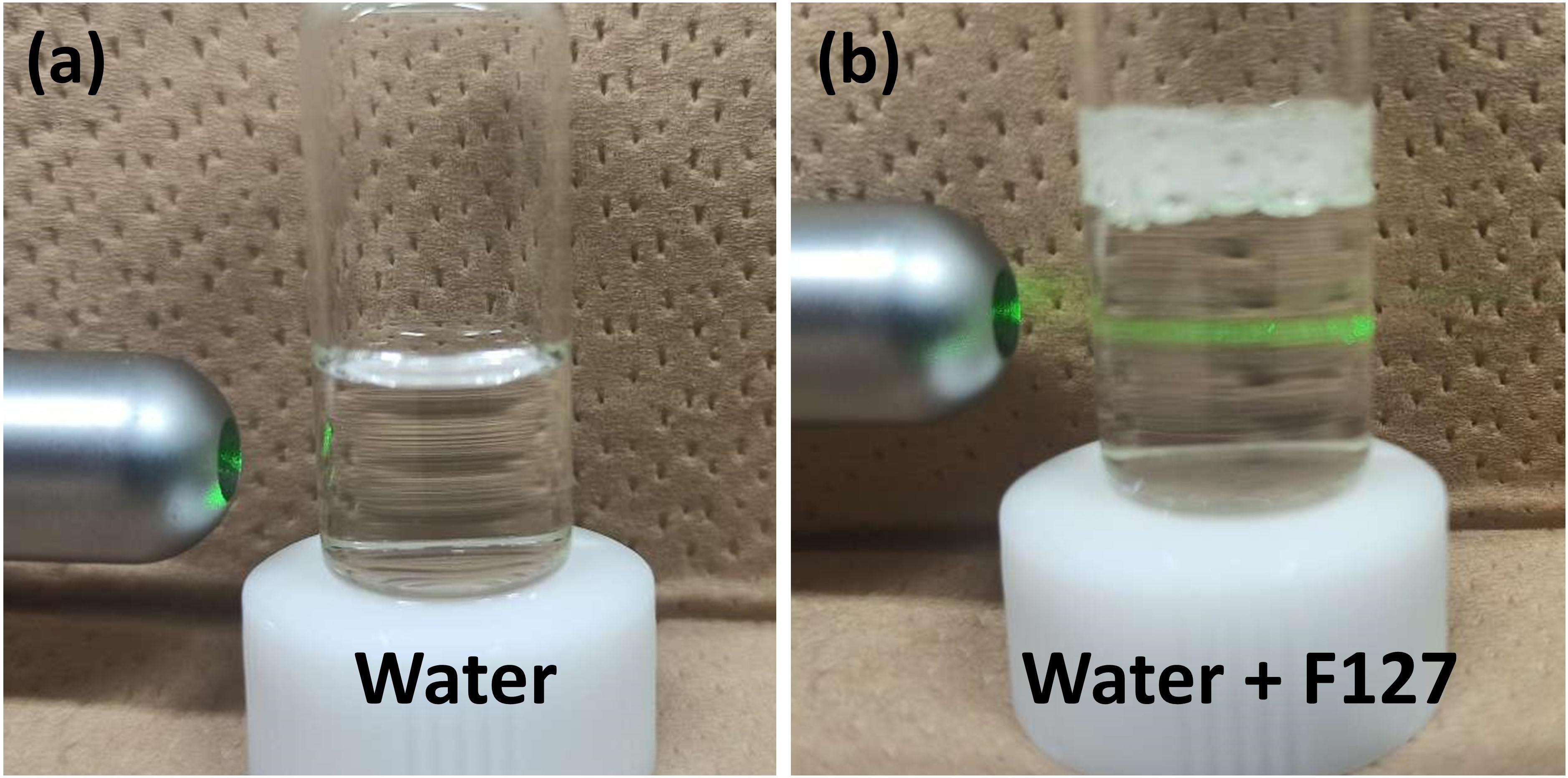
**

**Figure S3.** Optical photographs of laser light interacting with (a) milli Q water and (b) F127 polymer dissolved in milli Q water resulting in micellization process and the associated Tyndall effect.


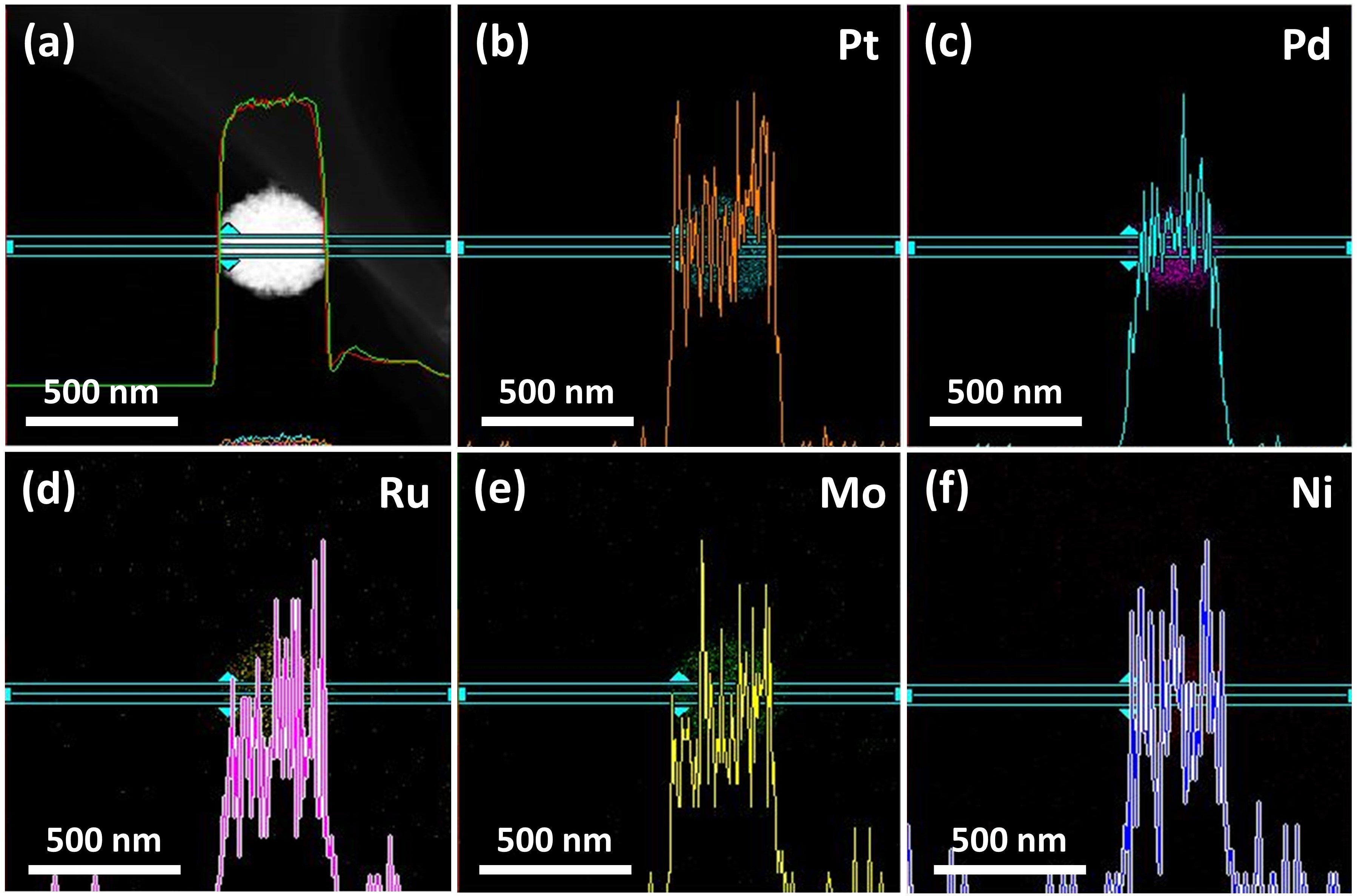


**Figure S4.**  (a) HAADF-STEM image of HEA10. (b-f) The corresponding elemental distribution profiles were obtained using the line scan collected with EDS.


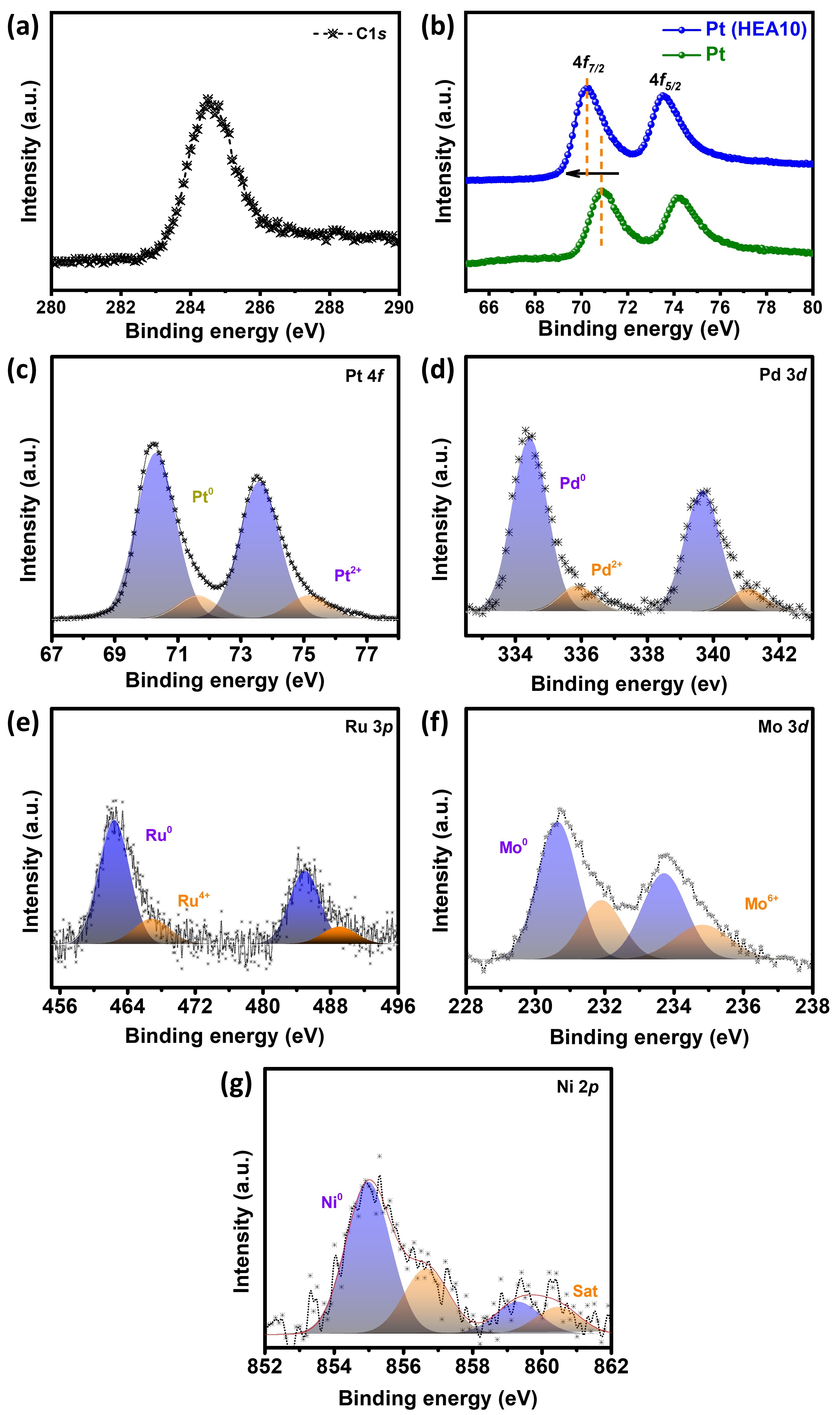


**Figure S5.** (a) C 1s XPS (HRXPS) spectrum from Pt-C. (b) HRXPS spectra of Pt 4*f* of HEA10 (PtPdRuMoNi mesoporous nanospheres) and Pt. (c-g) Deconvoluted HRXPS spectra for elemental valance state analysis of Pt 4*f*, Pd 3*d*, Ru 3*p*, Mo 3*d* and Ni 2*p* of HEA10.


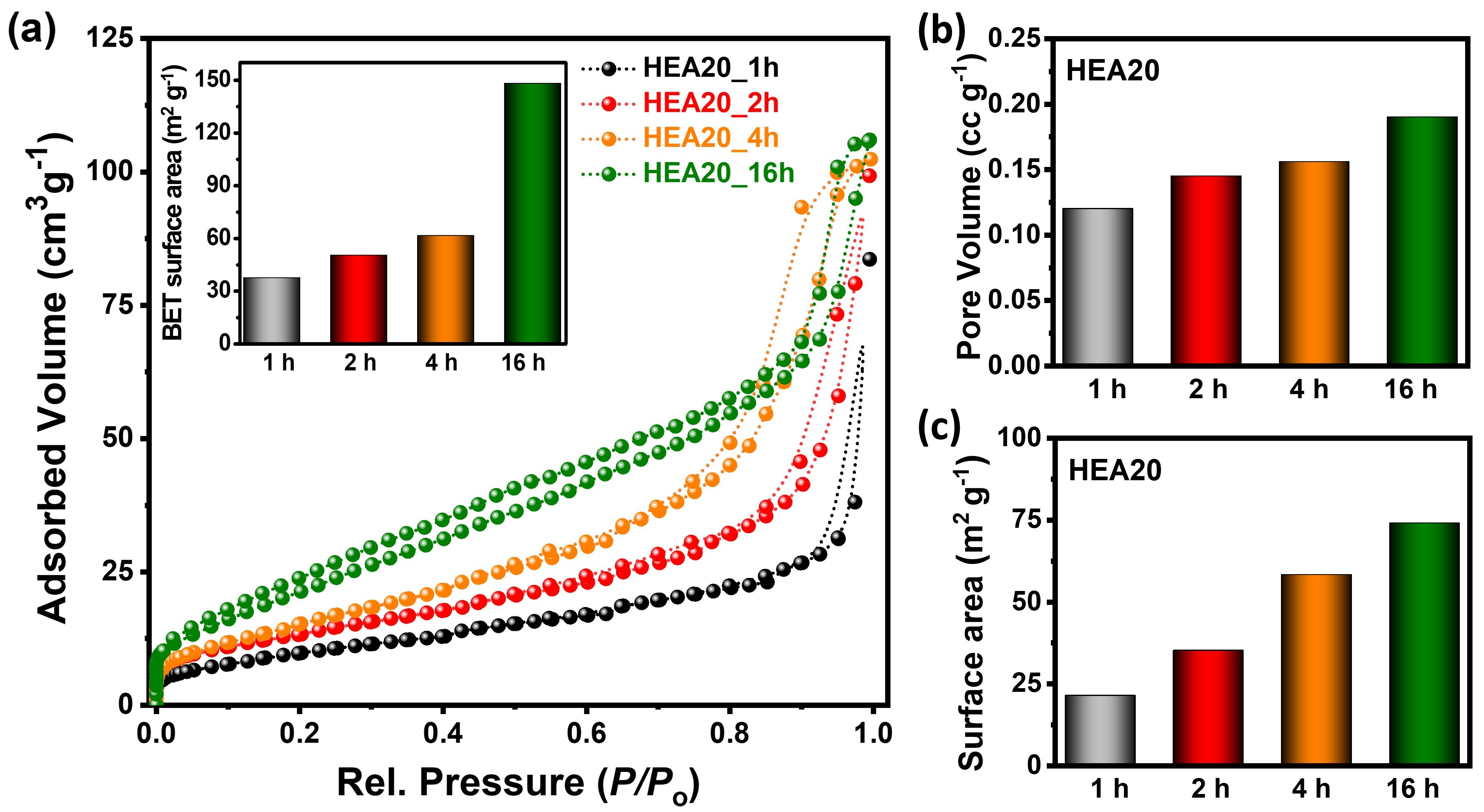


**Figure S6** (a) Nitrogen adsorption-desorption isotherms, (b) Pore volume, and (c) pore surface area for HEA20 mesoporous nanospheres for 1, 2 4, and 16 h reaction time. The inset of Figure a shows the corresponding specific surface area (BET).


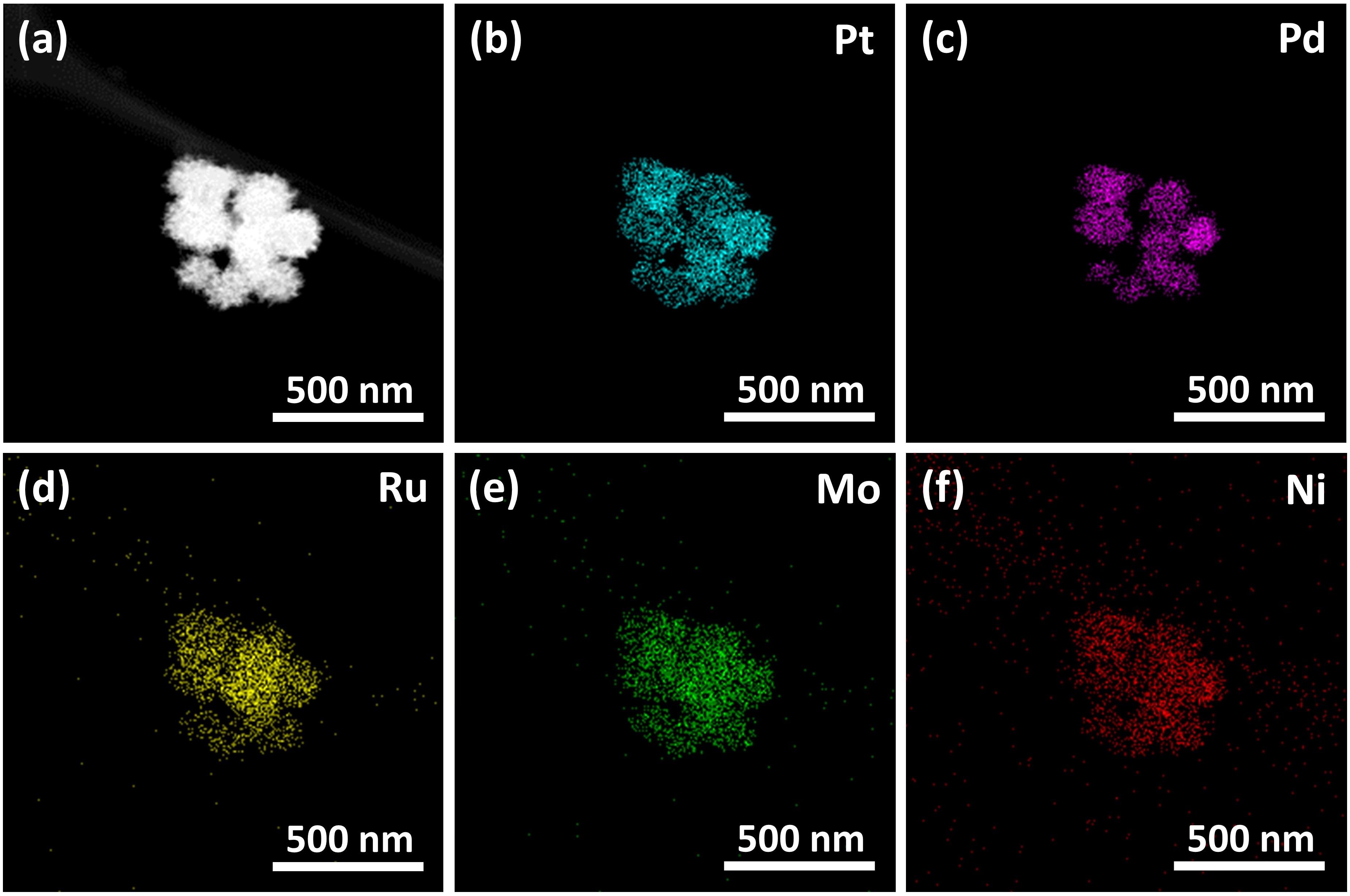


**Figure S7.**  (a) HAADF-STEM image of HEA20 mesoporous nanospheres. (b-f) Concomitant elemental mapping.

**
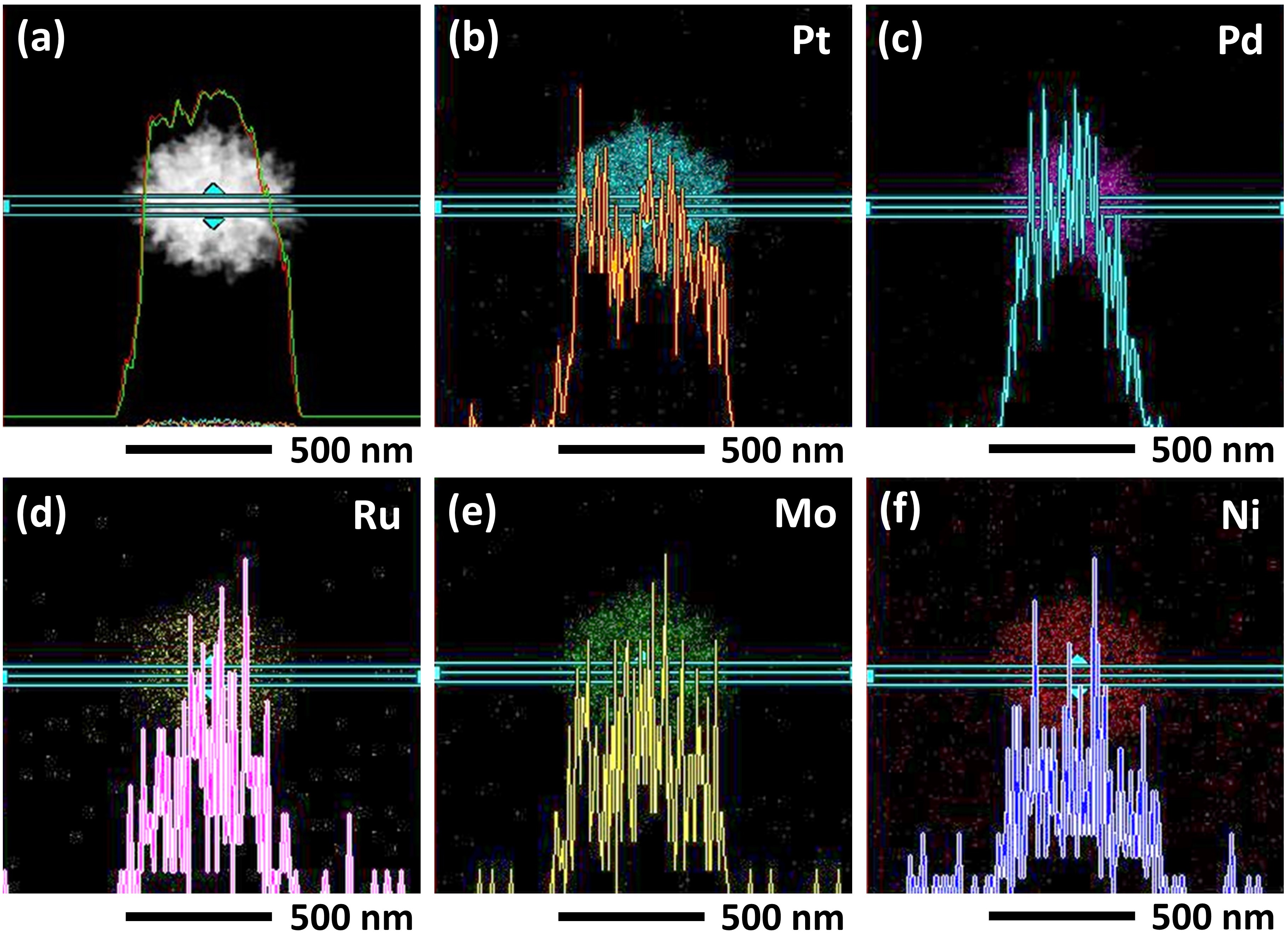
**

**Figure S8.** (a) HAADF-STEM image of HEA20. (b-f) The corresponding elemental distribution profiles were obtained using the line scan collected with EDS.


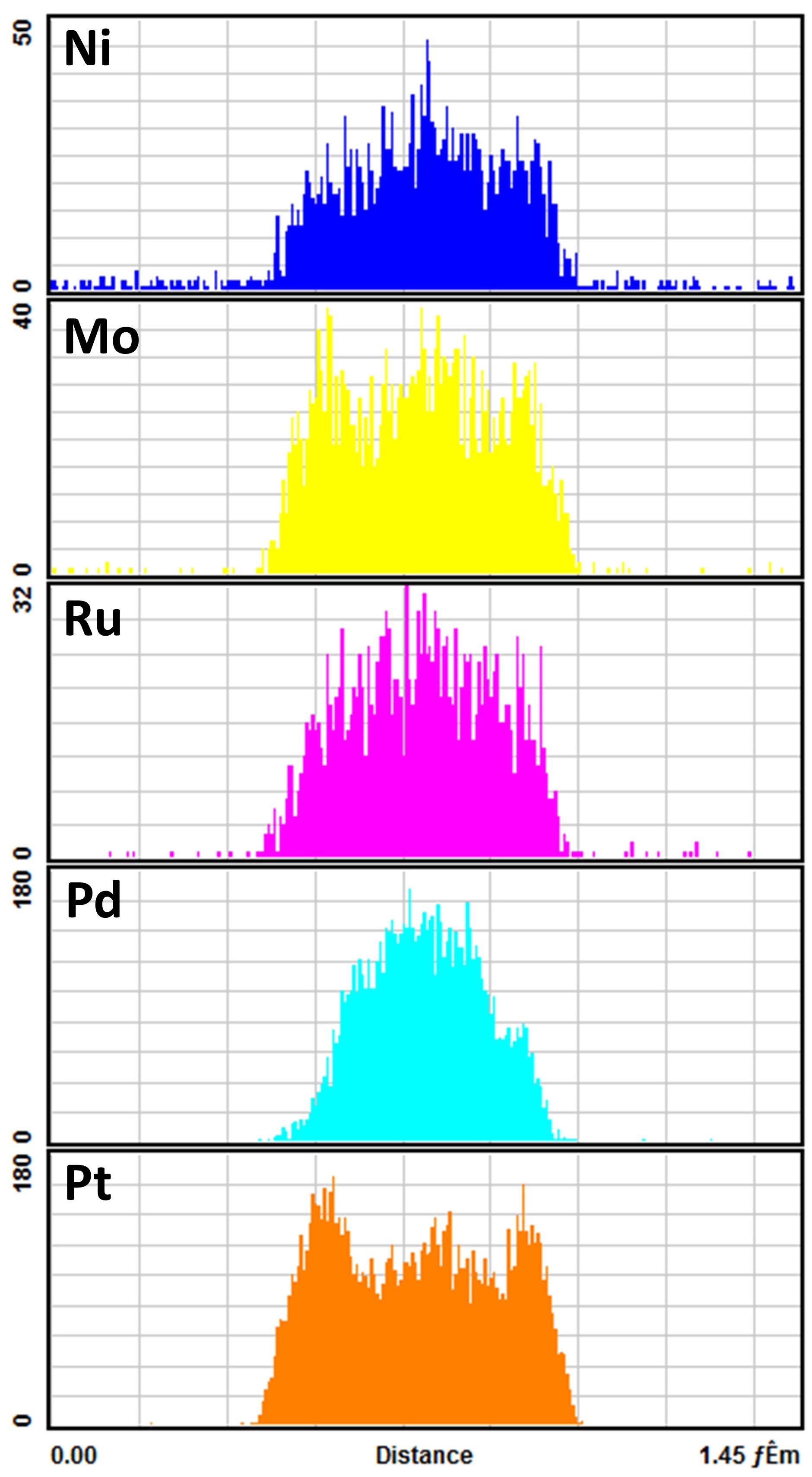


**Figure S9.** Elemental distribution profile in HEA20, deduced from elemental line scanning.


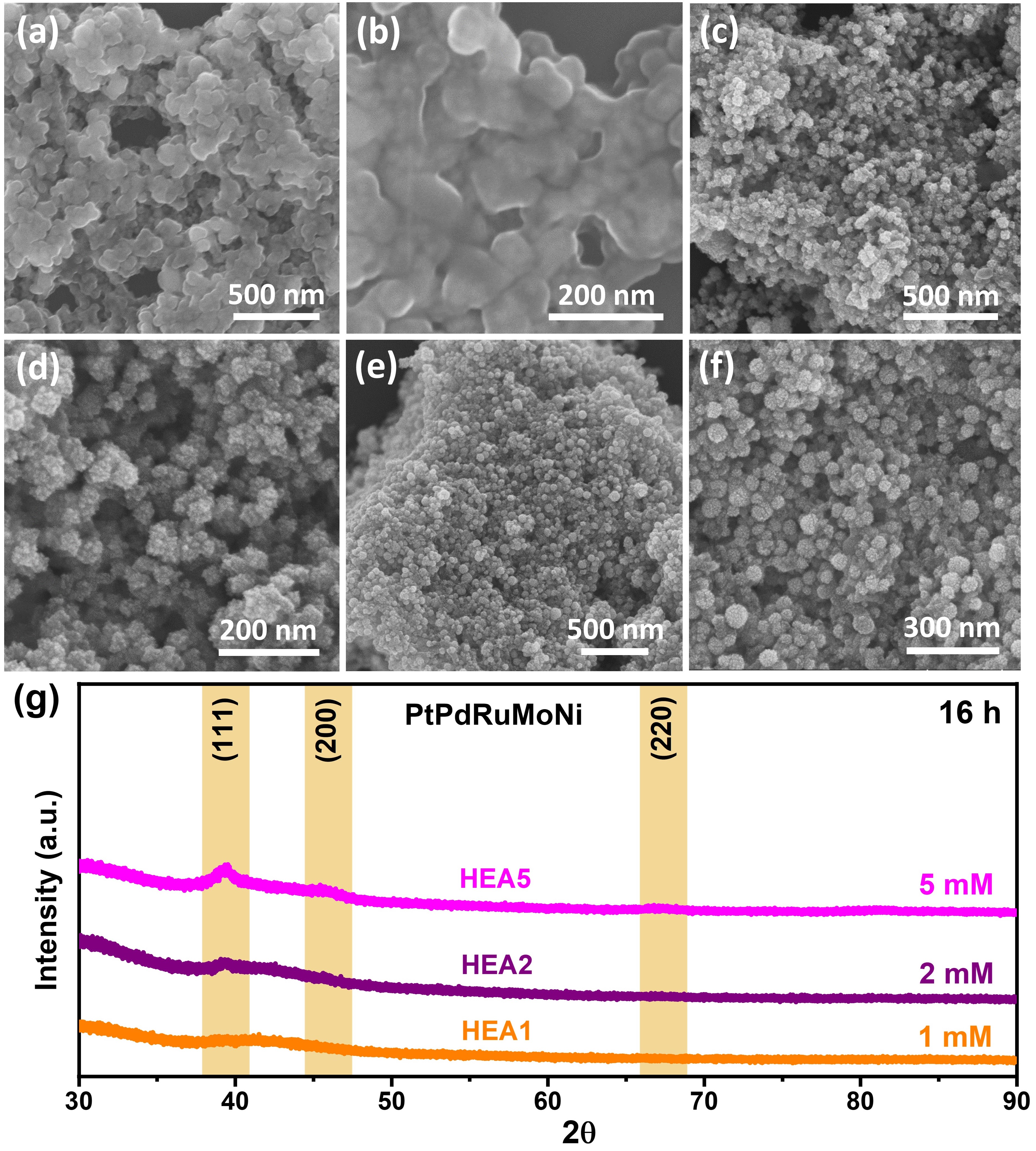


**Figure S10.** SEM images of (a,b) HEA1, (c,d) HEA2, and (e,f) HEA5 at different magnifications. (f) XRD patterns of HEA1, HEA2, and HEA5.


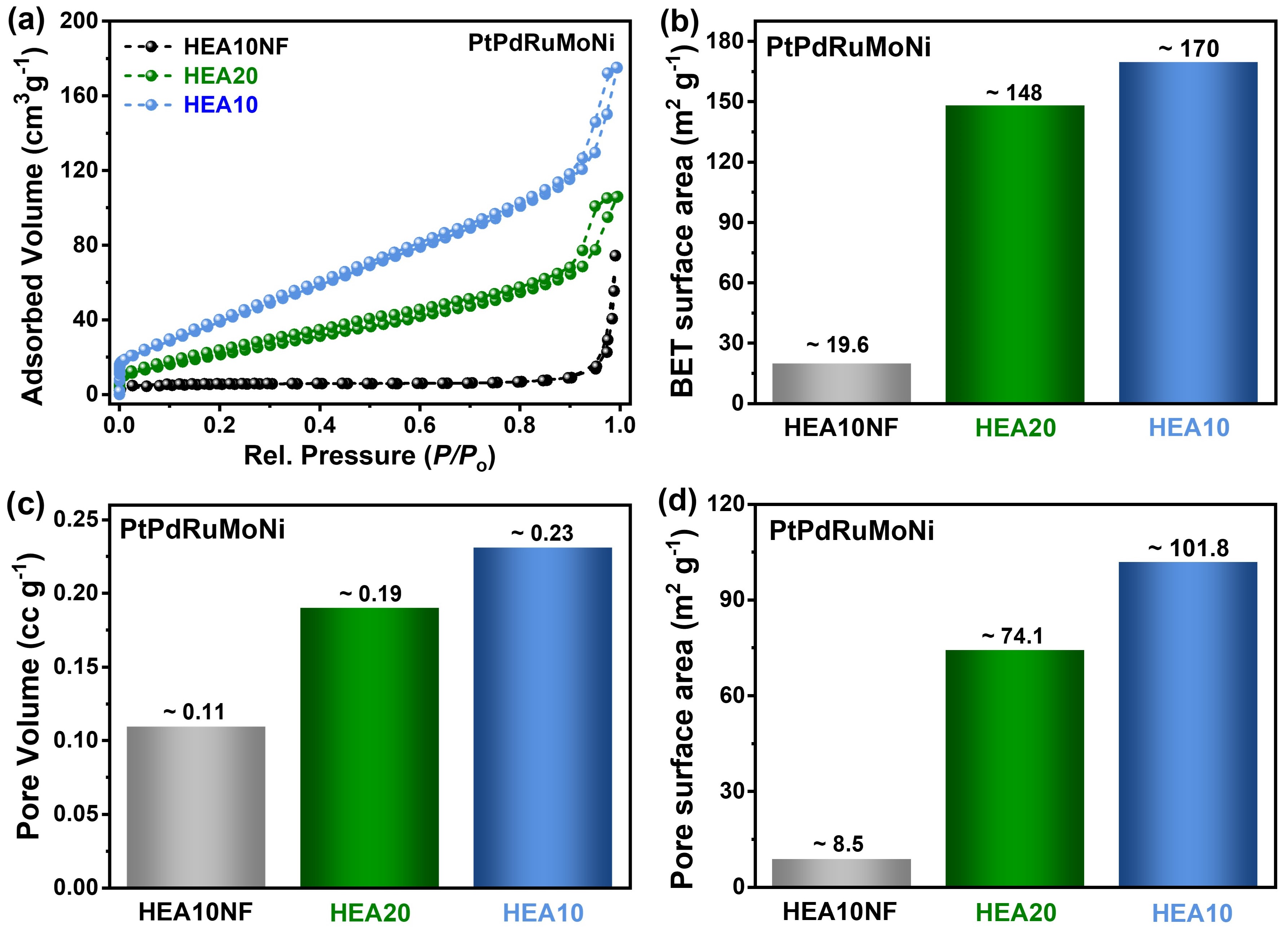


**Figure S11.** (a) Nitrogen adsorption-desorption isotherms on HEA10, HEA10NF, and HEA20. (b-d) The respective BET-specific surface area, pore volume, and pore surface area for HEA10, HEA10NF, and HEA20 are deduced from Figure a.

**
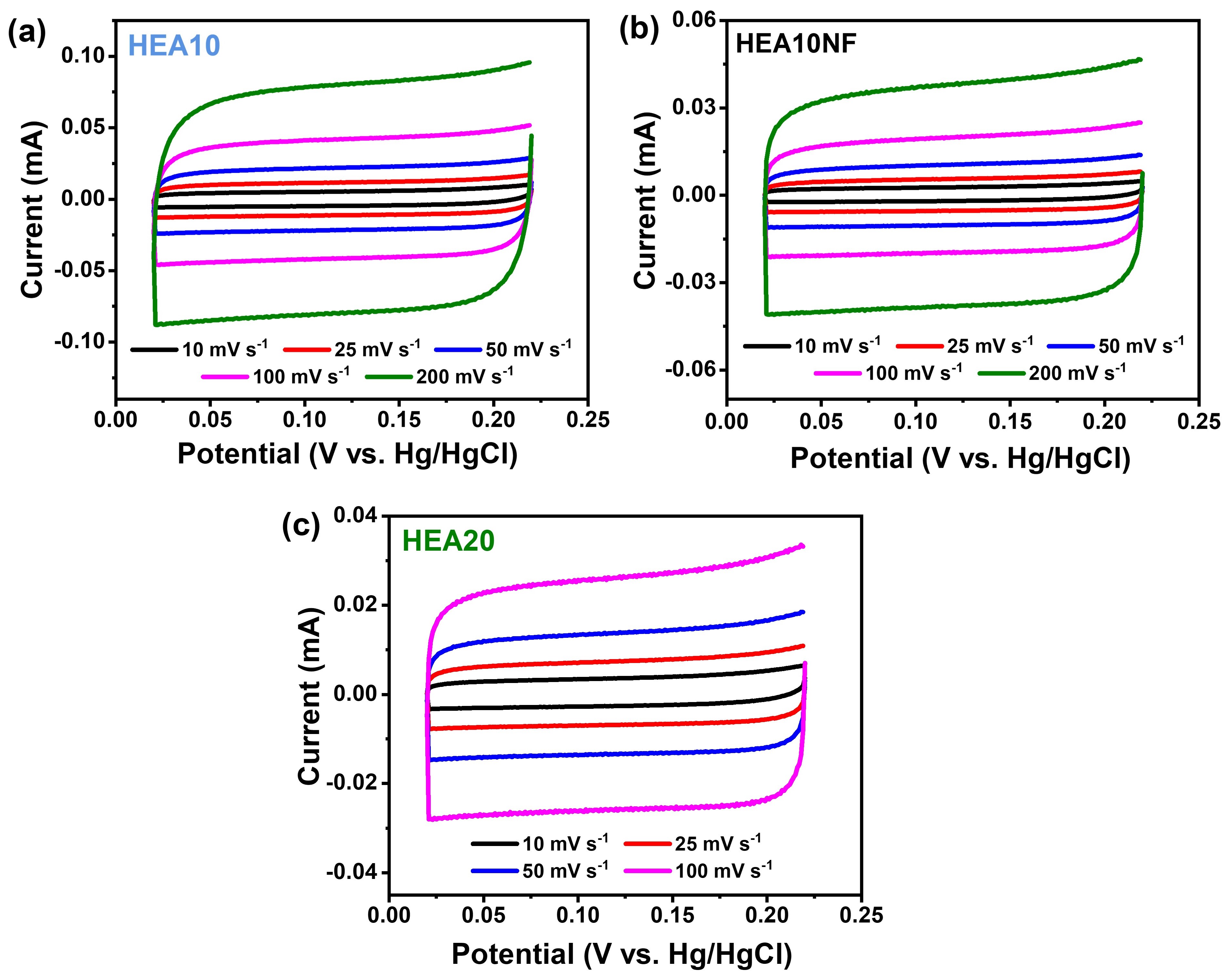
**

**Figure S12.** (a-c) CV curves on HEA10, HEA10NF, and HEA20, respectively, at different scan speeds in non-Faradic regions in a nitrogen-purged alkaline medium (0.1 M KOH). The double layer capacitance (*C*_dl_), a quantity directly related to the electrochemically active surface area, was evaluated from these CV curves for comparative studies and presented in Table S3.

**
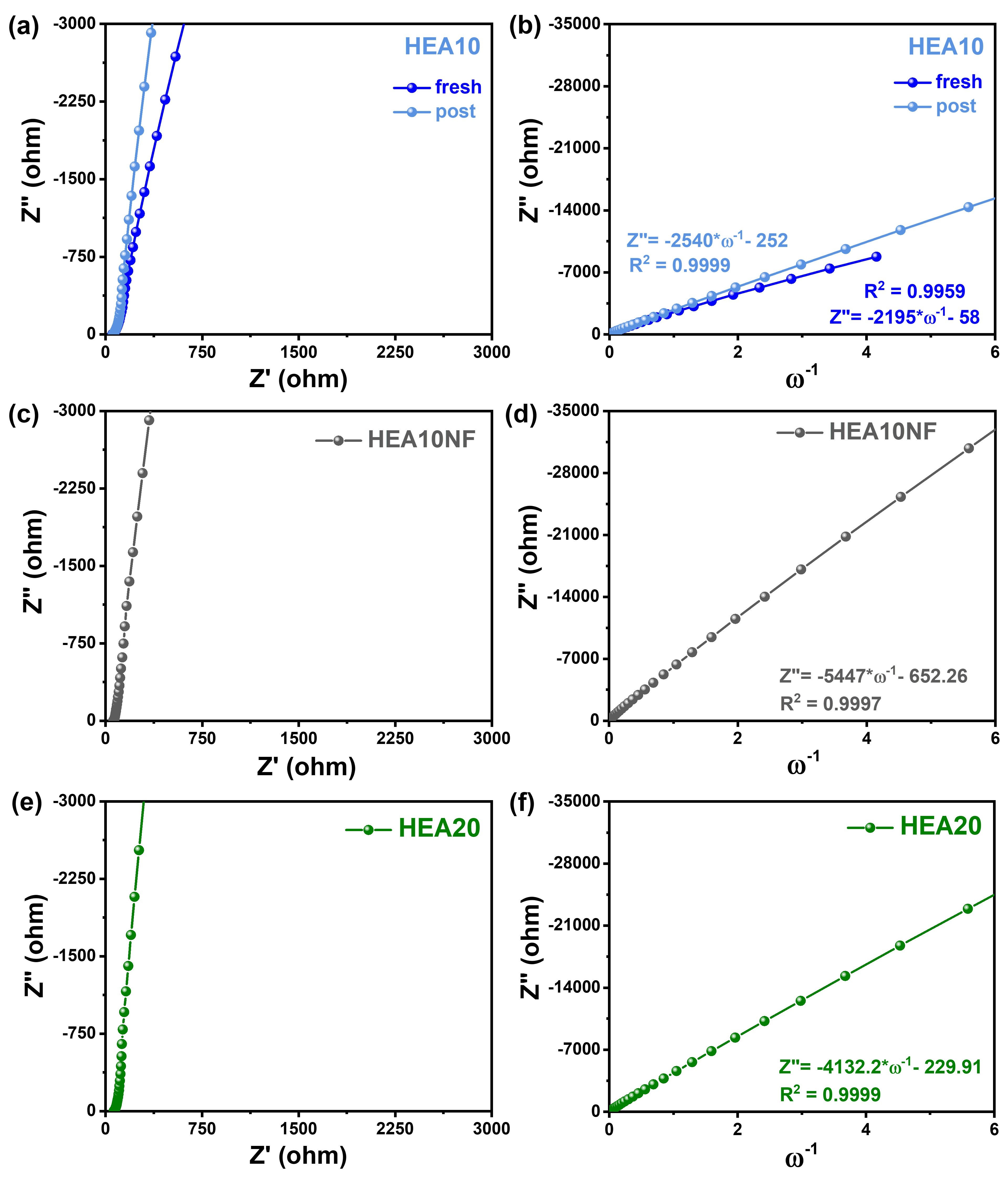
**

**Figure S13.** Electrochemical impedance spectroscopic measurements on (a,b) HEA10 (fresh and post electrochemical application), (c,d) HEA10NF, and (e,f) HEA20 in an alkaline medium (0.1 M KOH) to estimate the concomitant *C*_dl_ values (Table S4).

**
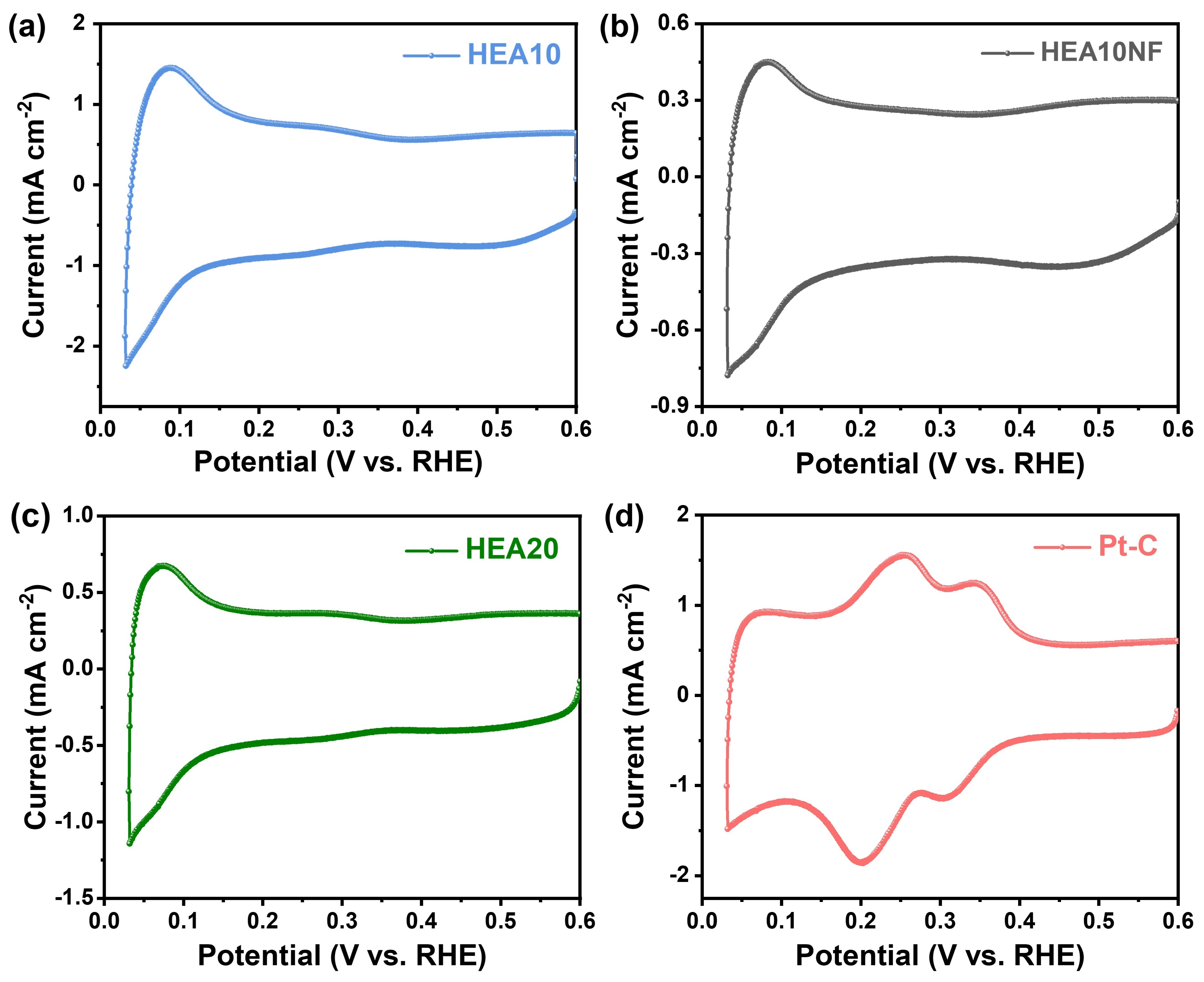
**

**Figure S14.** Hydrogen underpotential deposition (HUPD) CV profiles on (a) HEA10, (b) HEA10NF, (c) HEA20, and (d) commercial Pt-C in an alkaline medium (0.1 M KOH) to evaluate the concomitant *C*_dl_ values (Table S5).


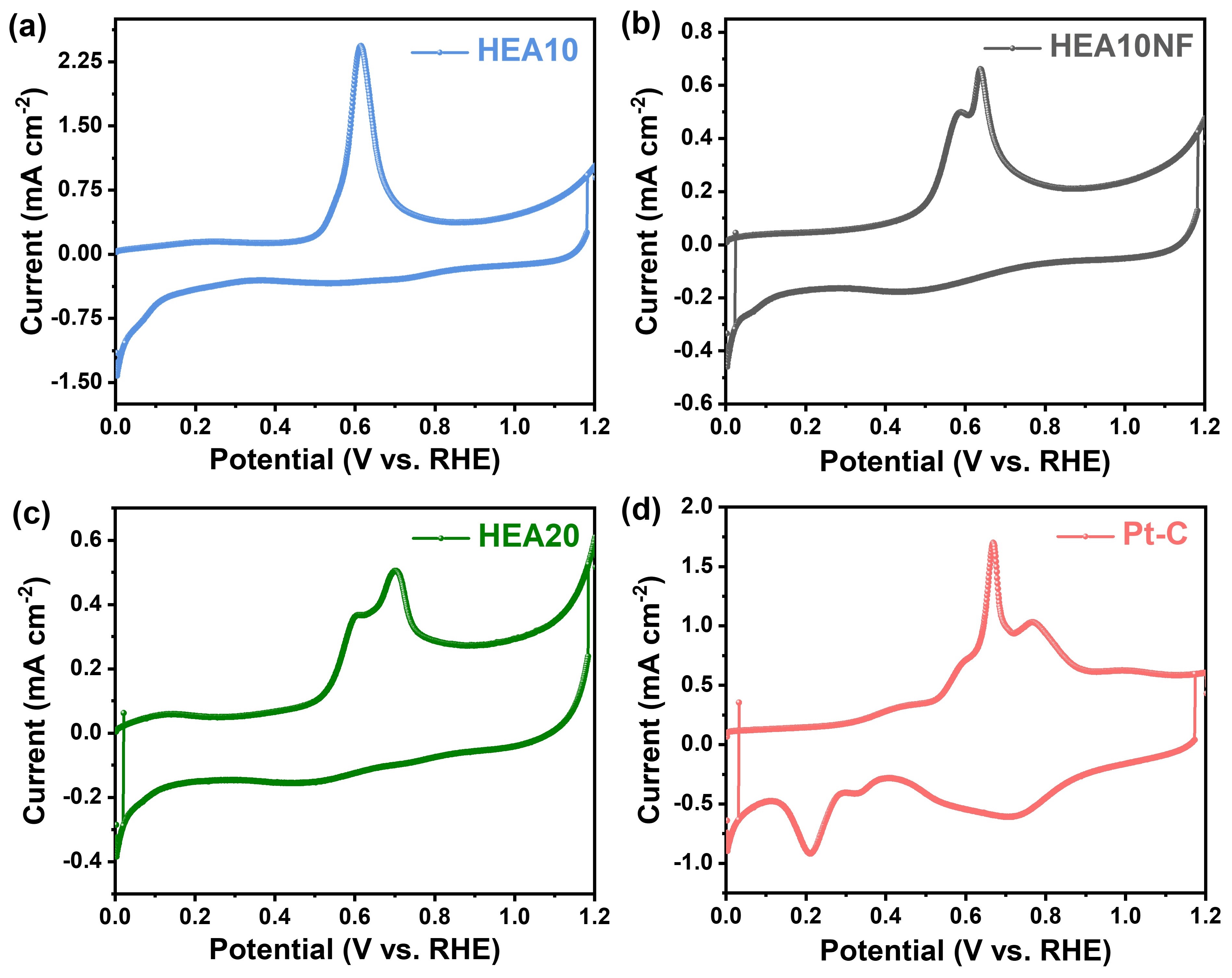


**Figure S15.** CO striping profiles on (a) HEA10, (b) HEA10NF, (c) HEA20, and (d) Pt-C in an alkaline medium (0.1 M KOH) at a potential scan speed of 20 mV s^−1^ to evaluate the concomitant *C*_dl_ values (Table S6).

**
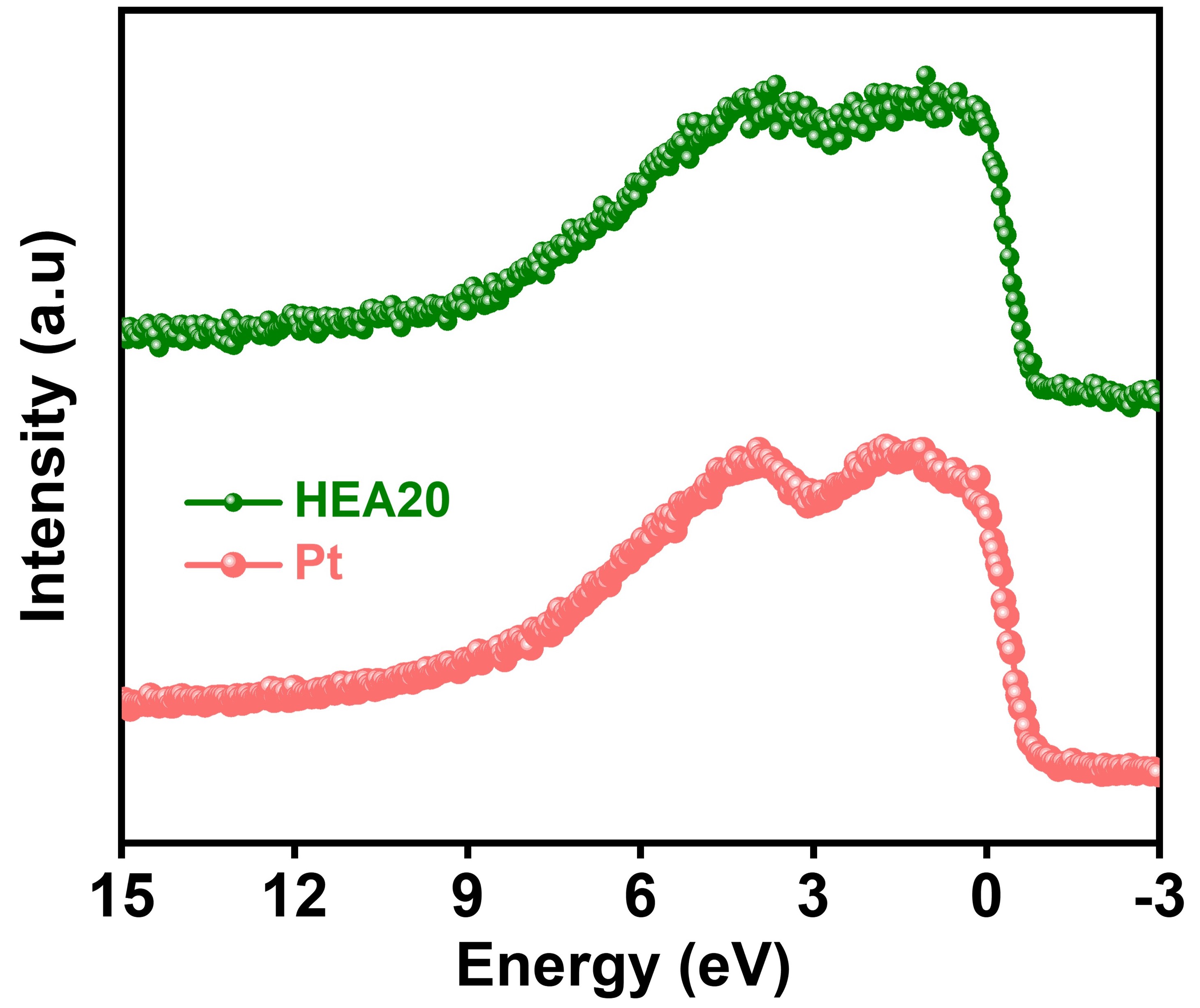
**

**Figure S16.** The HAXPES spectra show the valance band of HEA20 and Pt using a Cr Ka source.

**
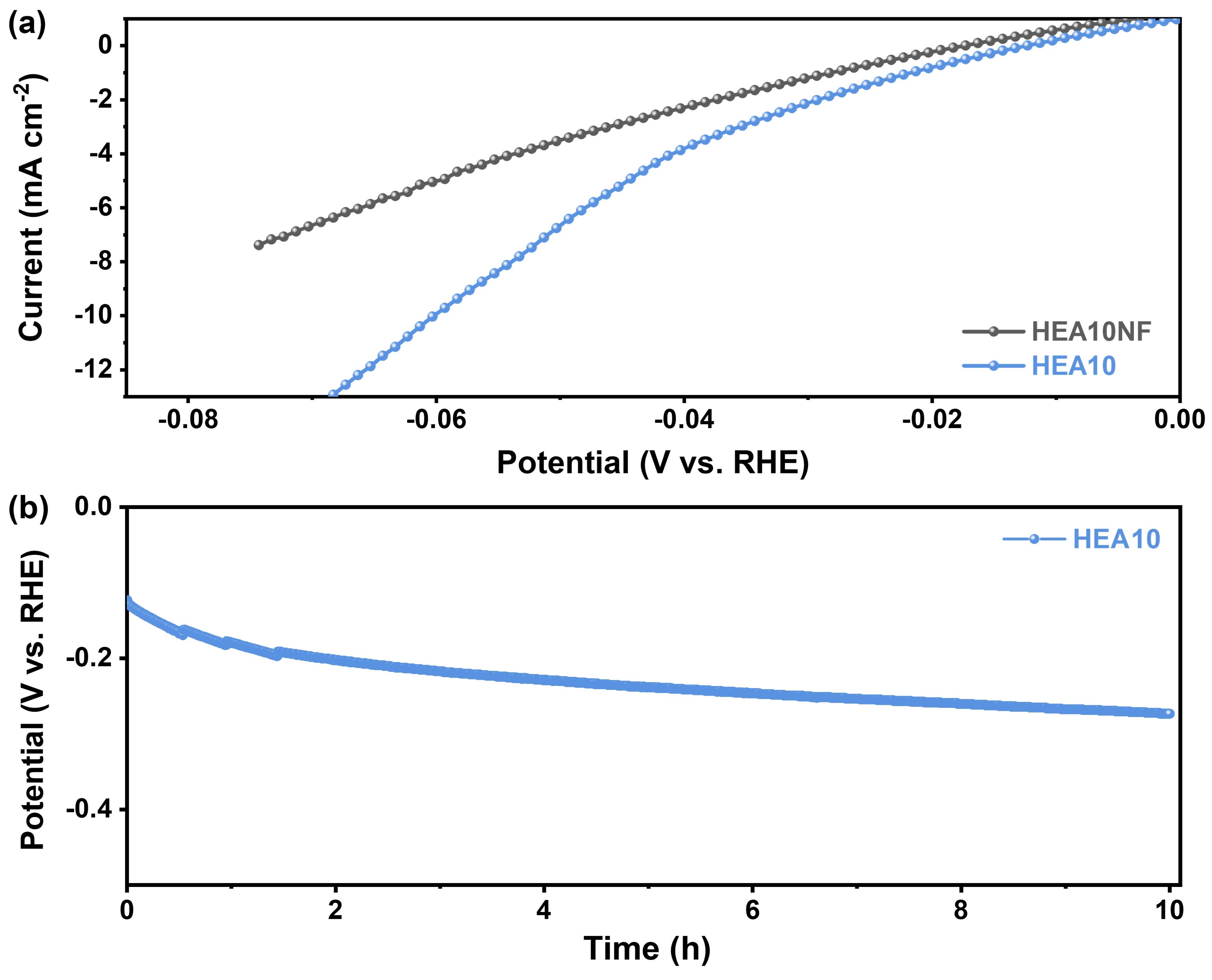
**

**Figure S17.** (a) HER-LSV polarization curves on HEA10NF and HEA10 in 0.1 M KOH solution (*ω* = 1600 rpm, *v* = 10 mV s^−1^). (b) Stability study on HEA10 @10 mA cm^−2^.

**
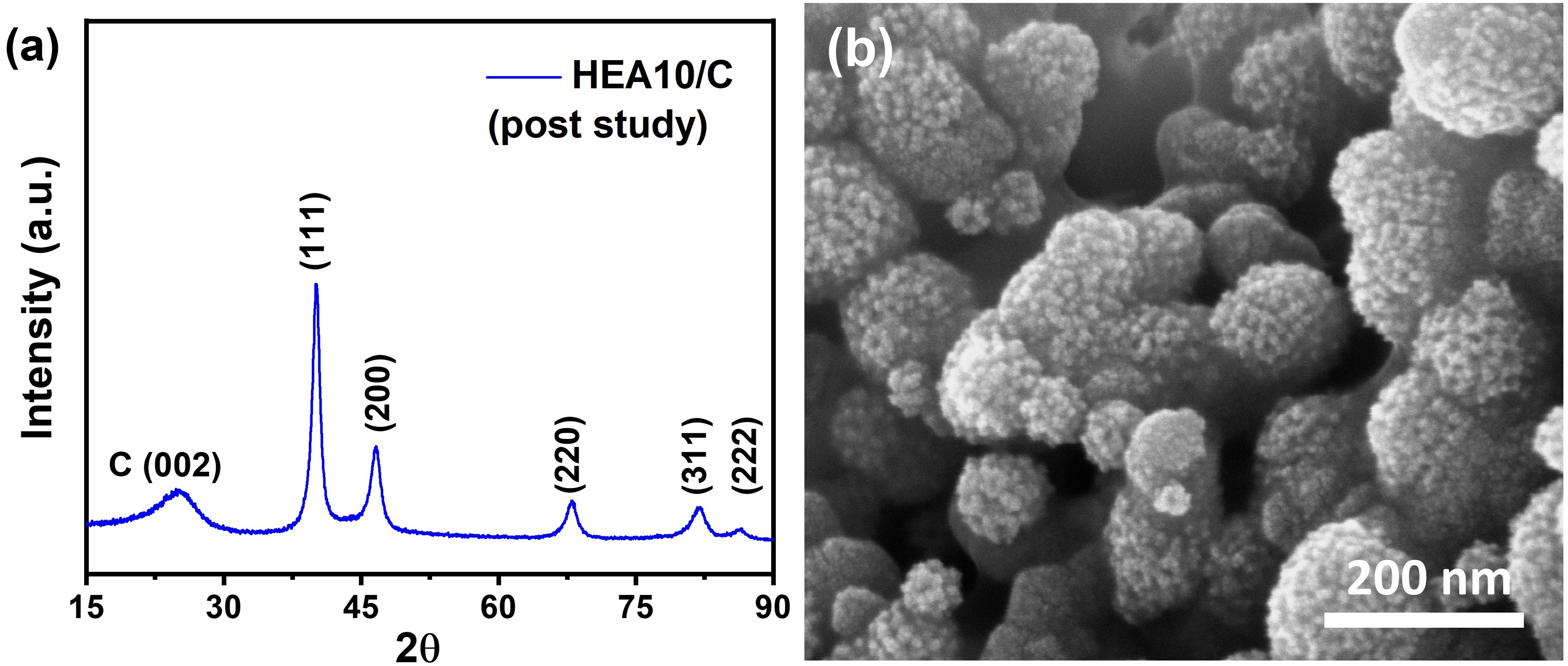
**

**Figure S18.**  (a) XRD pattern and (b) SEM image of HEA10 post electrochemical studies. The presence of an extra peak around 25°is attributed to the presence of carbon, which was used during catalyst ink preparation.

**
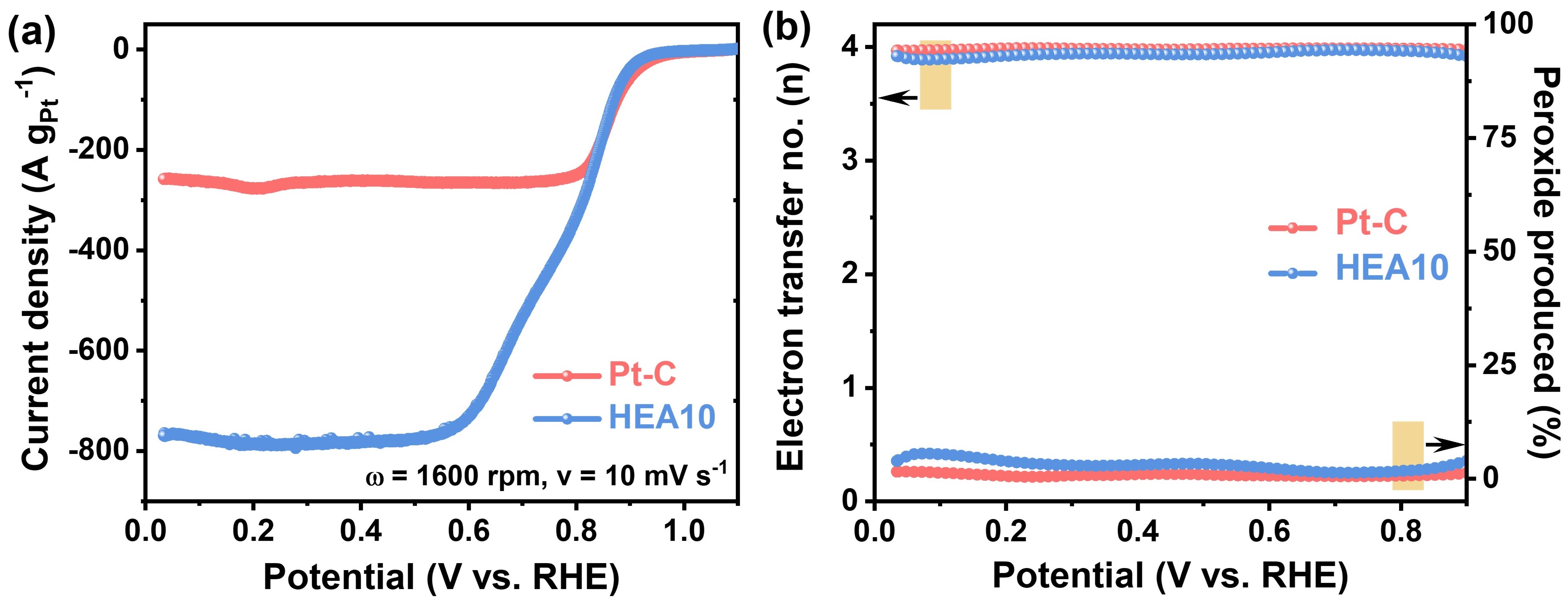
Figure S19.** (a) Pt mass normalized ORR-LSV polarization curves. (b) Peroxide generation and no. of electrons transfer involved during ORR process on HEA10 and Pt-C in oxygen-purged 0.1 M KOH aqueous solution (*w* = 1600 rpm, *v* = 10 mV s^−1^).

**Table S1.** The comparative HER performance of some of the recently reported multi-metallic and HEA nanosystems in the alkaline medium.

| **Electrocatalyst** | **Electrolyte** | **Overpotential (mV)**  **@ 10 mA cm**^−^**^2^** | **Reference** |
| --- | --- | --- | --- |
| PtPdRhRuCu | 1 M KOH | 10 | [1] |
| MoSe_2_/MoO_3_ | 0.1 M KOH | 270 | [2] |
| FeCoNiCuMnN/CC400 | 1 M KOH | 184 | [3] |
| CoZnCdCuMnS | 1 M KOH | 173 | [4] |
| Ni_20_Fe_20_Mo_10_Co_35_Cr_15_ | 0.1 M KOH | 172 | [5] |
| PtCuNi/CNF@CF | 1 M KOH | 150 (@5 mA cm^−2^) | [6] |
| CoNiCuMgZn/graphene | 1 M KOH | 158 | [7] |
| CoCrMnNiFeP | 1 M KOH | 136 | [8] |
| PtCoFe@CN | 1 M KOH | 120 | [9] |
| MoNiN/C | 0.1 M KOH | 110 | [10] |
| 1.08 wt% Pt/N-Mo_2_C | 1 M KOH | 100 | [11] |
| AlCrFeCoNiW | 1 M KOH | 101 | [12] |
| Co(OH)_2_@PdNi HNSs/NF | 1 M NaOH | 90 | [13] |
| PtNi_frame/Ni(OH)_2_ | 0.1 M KOH | ~63 (@ 5 mA cm^−2^) | [14] |
| PtNi_3_/C NF | 0.1 M KOH | ~88 (@ 5 mA cm^−2^) |  |
| PtNiNi(OH)_2_ | 0.1 M KOH | ~110 (@ 5 mA cm^−2^) |  |
| Au_33_Pt_67_ NPs | 0.1 M KOH | 88 | [15] |
| FeCoNiAlTi | 1 M KOH | 88.2 | [16] |
| NiN-C250/Pt | 1 M KOH | 83.5 | [17] |
| FeNiCoMnVOx | 1 M KOH | 81 | [18] |
| PdPtS | 1 M KOH | 71 | [19] |
| PtNi alloy | 0.1 M KOH | 82 | [20] |
| PtNi/C | 0.1 M KOH | 70 | [21] |
| Co_0.6_(VMnNiZn)_0.4_PS3 | 1 M KOH | 65.9 | [22] |
| Pt/Fe-NF | 0.1 KOH | 65 | **[20]** |
| hcp-excavated-PtNi | 0.1 M KOH | 65 | [23] |
| Pt_3_Ni_3_ NWs/C-air | 0.1 M KOH | ~70 (@ 5 mA cm^−2^) | [24] |
| HEA 10  (PtPdRuMoNi) | 0.1 M KOH | 60 | **This work** |

| **Wt. %** | **HEA10** | **HEA20** | **HEA10NF** |
| --- | --- | --- | --- |
| Pt | 34 | 40 | 31 |
| Pd | 31 | 39 | 31 |
| Ru | 21 | 13 | 19 |
| Mo | 9 | 4.8 | 14 |
| Ni | 5 | 3.2 | 5 |

**Table S2.** Elemental composition analysis of HEA10, HEA20, and HEA10NF using inductively coupled plasma optical emission spectroscopy (ICP-OES).

**Table S3.** The *C*_dl_ (F g^−1^) values for HEA10, HEA10NF, and HEA20 were determined from CV measurements (Figure S12).

| ***C*_dl__ (F g**^−1^**)** | |
| --- | --- |
| HEA10 | 26.29 |
| HEA20 | 16.56 |
| HEA10NF | 12.41 |

**Table S4.** The *C*_dl_ (F g^−1^) values for HEA10, HEA10NF, and HEA20 are determined from EIS measurements (Figure S13).

| ***C*_dl__ (F g**^−1^**)** | |
| --- | --- |
| HEA10 | 33.5 |
| HEA20 | 18.10 |
| HEA10NF | 13.9 |

**Table S5.** The *C*_dl_ (F g^−1^) values for HEA10, HEA10NF, and HEA20 were determined from HUPD measurements (Figure S14).

| ***C*_dl__ (F g**^−1^**)** | |
| --- | --- |
| HEA10 | 36.48 |
| HEA20 | 11.46 |
| HEA10NF | 6.59 |
| Pt-C | 9.76 |

**Table S6.** The *C*_dl_ (F g^−1^) values for HEA10, HEA10NF, and HEA20 were determined from CO-stripping measurements (Figure S15).

| ***C*_dl__ (F g**^−1^**)** | |
| --- | --- |
| HEA10 | 75.24 |
| HEA20 | 24 |
| HEA10NF | 24.62 |
| Pt-C | 46.44 |

**Table S7.** The *C*_dl_ (F g^−1^) values for HEA10 before and after the electrochemical study are determined from EIS measurements (Figure S13a, b).

| ***C*_dl__ (F g**^−1^**)** | |
| --- | --- |
| Fresh | 33.5 |
| Post-study | 29.4 |

**References**

[1] Y. Kang, O. Cretu, J. Kikkawa, K. Kimoto, H. Nara, A. S. Nugraha, H. Kawamoto, M. Eguchi, T. Liao, Z. Sun, T. Asahi, Y. Yamauchi, *Nat. Commun.* **2023**, *14*, 1.

[2] Q. Zhou, G. Zhao, K. Rui, Y. Chen, X. Xu, S. X. Dou, W. Sun, *Nanoscale* **2019**, *11*, 717.

[3] C. Liu, H. Zhu, S. Lu, F. Duan, M. Du, *New J. Chem.* **2021**, *45*, 22255.

[4] Y. Lei, L. Zhang, W. Xu, C. Xiong, W. Chen, X. Xiang, B. Zhang, H. Shang, *Nano Res.* **2022**, *15*, 6054.

[5] G. Zhang, K. Ming, J. Kang, Q. Huang, Z. Zhang, X. Zheng, X. Bi, *Electrochim. Acta* **2018**, *279*, 19.

[6] Y. Shen, A. C. Lua, J. Xi, X. Qiu, *ACS Appl. Mater. Interfaces* **2016**, *8*, 3464.

[7] D. Feng, Y. Dong, P. Nie, L. Zhang, Z. A. Qiao, *Chem. Eng. J.* **2022**, *430*, 132883.

[8] X. Zhao, Z. Xue, W. Chen, Y. Wang, T. Mu, *ChemSusChem* **2020**, *13*, 2038.

[9] J. Chen, Y. Yang, J. Su, P. Jiang, G. Xia, Q. Chen, *ACS Appl. Mater. Interfaces* **2017**, *9*, 3596.

[10] F. Wang, Y. Sun, Y. He, L. Liu, J. Xu, X. Zhao, G. Yin, L. Zhang, S. Li, Q. Mao, Y. Huang, T. Zhang, B. Liu, *Nano Energy* **2017**, *37*, 1.

[11] Y. Qiu, Z. Wen, C. Jiang, X. Wu, R. Si, J. Bao, Q. Zhang, L. Gu, J. Tang, X. Guo, *Small* **2019**, *15*, 1900014.

[12] X. Han, Q. Chen, Q. Chen, Q. Wu, Z. Xu, T. Zheng, W. Li, D. Cui, Z. Duan, J. Zhang, J. Li, H. Li, Z. Wang, J. Wang, Z. Xia, *J. Mater. Chem. A* **2022**, *10*, 11110.

[13] J. X. Feng, L. X. Ding, S. H. Ye, X. J. He, H. Xu, Y. X. Tong, G. R. Li, *Adv. Mater.* **2015**, *27*, 7051.

[14] C. Chen, Y. Kang, Z. Huo, Z. Zhu, W. Huang, H. L. Xin, J. D. Snyder, D. Li, J. A. Herron, M. Mavrikakis, M. Chi, K. L. More, Y. Li, N. M. Markovic, G. A. Somorjai, P. Yang, V. R. Stamenkovic, *Science* **2014**, *343*, 1339.

[15] W. Wu, Z. Tang, K. Wang, Z. Liu, L. Li, S. Chen, *Electrochim. Acta* **2018**, *260*, 168.

[16] Z. Jia, T. Yang, L. Sun, Y. Zhao, W. Li, J. Luan, F. Lyu, L. C. Zhang, J. J. Kruzic, J. J. Kai, J. C. Huang, J. Lu, C. T. Liu, *Adv. Mater.* **2020**, *32*, 2000385.

[17] P. Li, G. Zhao, P. Cui, N. Cheng, M. Lao, X. Xu, S. X. Dou, W. Sun, *Nano Energy* **2021**, *83*, 105850.

[18] S. Ding, Y. Sun, F. Lou, L. Yu, B. Xia, J. Duan, Y. Zhang, S. Chen, *J. Power Sources* **2022**, *520*, 230873.

[19] J. Fan, K. Qi, L. Zhang, H. Zhang, S. Yu, X. Cui, *ACS Appl. Mater. Interfaces* **2017**, *9*, 18008.

[20] C. Zhang, B. Chen, D. Mei, X. Liang, *J. Mater. Chem. A* **2019**, *7*, 5475.

[21] R. Kavian, S. Il Choi, J. Park, T. Liu, H. C. Peng, N. Lu, J. Wang, M. J. Kim, Y. Xia, S. W. Lee, *J. Mater. Chem. A* **2016**, *4*, 12392.

[22] R. Wang, J. Huang, X. Zhang, J. Han, Z. Zhang, T. Gao, L. Xu, S. Liu, P. Xu, B. Song, *ACS Nano* **2022**, *16*, 3593.

[23] Z. Cao, Q. Chen, J. Zhang, H. Li, Y. Jiang, S. Shen, G. Fu, B. A. Lu, Z. Xie, L. Zheng, *Nat. Commun.* **2017**, *8*, 1.

[24] P. Wang, K. Jiang, G. Wang, J. Yao, X. Huang, *Angew. Chem. Int. Ed.* **2016**, *55*, 12859.
